# Digital Patient Experience: Umbrella Systematic Review

**DOI:** 10.2196/37952

**Published:** 2022-08-04

**Authors:** Tingting Wang, Guido Giunti, Marijke Melles, Richard Goossens

**Affiliations:** 1 Industrial Design Engineering Delft University of Technology Delft Netherlands; 2 Digital Health Design and Development University of Oulu Oulu Finland

**Keywords:** digital health, eHealth, telemedicine, telehealth, mobile health, mHealth, patient experience, user experience, influencing factors, user-centered design, human-computer interaction

## Abstract

**Background:**

The adoption and use of technology have significantly changed health care delivery. Patient experience has become a significant factor in the entire spectrum of patient-centered health care delivery. Digital health facilitates further improvement and empowerment of patient experiences. Therefore, the design of digital health is served by insights into the barriers to and facilitators of digital patient experience (PEx).

**Objective:**

This study aimed to systematically review the influencing factors and design considerations of PEx in digital health from the literature and generate design guidelines for further improvement of PEx in digital health.

**Methods:**

We performed an umbrella systematic review following the PRISMA (Preferred Reporting Items for Systematic Reviews and Meta-Analyses) methodology. We searched Scopus, PubMed, and Web of Science databases. Two rounds of small random sampling (20%) were independently reviewed by 2 reviewers who evaluated the eligibility of the articles against the selection criteria. Two-round interrater reliability was assessed using the Fleiss-Cohen coefficient (*k*1=0.88 and *k*2=0.80). Thematic analysis was applied to analyze the extracted data based on a small set of a priori categories.

**Results:**

The search yielded 173 records, of which 45 (26%) were selected for data analysis. Findings and conclusions showed a great diversity; most studies presented a set of themes (19/45, 42%) or descriptive information only (16/45, 36%). The digital PEx–related influencing factors were classified into 9 categories: patient capability, patient opportunity, patient motivation, intervention technology, intervention functionality, intervention interaction design, organizational environment, physical environment, and social environment. These can have three types of impacts: positive, negative, or double edged. We captured 4 design constructs (personalization, information, navigation, and visualization) and 3 design methods (human-centered or user-centered design, co-design or participatory design, and inclusive design) as design considerations.

**Conclusions:**

We propose the following definition for digital PEx: “Digital patient experience is the sum of all interactions affected by a patient’s behavioral determinants, framed by digital technologies, and shaped by organizational culture, that influence patient perceptions across the continuum of care channeling digital health.” In this study, we constructed a design and evaluation framework that contains 4 phases—define design, define evaluation, design ideation, and design evaluation—and 9 design guidelines to help digital health designers and developers address digital PEx throughout the entire design process. Finally, our review suggests 6 directions for future digital PEx–related research.

## Introduction

### Background

Recently, there has been a significant increase in the use of digital health technologies. In addition, many countries currently use digital health technologies to support health care service delivery to overcome the disruptions caused by the COVID-19 pandemic. These include web-based patient consultations and requesting pharmacy and medication refills [1]. Digital health offers care without the risk of exposure to the virus, especially for vulnerable patients such as older adults and patients with chronic diseases [2]. Before the COVID-19 pandemic, there was increasing recognition of the potential of digital health to improve the accessibility of health care in different clinical settings (eg, ambulatory care, acute care, and inpatient care) [3]. Digital health provides an opportunity to both reduce the costs of care and improve patient affordability [4,5], and previous research suggests that digital health has the potential to provide health prevention, consultation, treatment, and management [5-10]. With digital health solutions continuing to grow in both number and functionality, patient interest in digital health has rapidly increased, leading to an expanding reliance on digital health technologies [11].

As digital health has become a more familiar term, it has generated many definitions, and the concept has been expanded to encompass a much broader set of scientific concepts and technologies [12]. These include digital health applications, ecosystems and platforms [13], patient portals [14], mobile health apps [15], eHealth records, and appointment scheduling applications [16]. For the purposes of this study, we will use eHealth, mobile health, telemedicine, telehealth, virtual health, remote health, electronic consultations, and health information systems (HISs) as interchangeable terms for digital health.

### Patient Experience in Digital Health

Digital health has the potential to improve patients’ overall health care experience [17-19]. However, there is currently no common concept for describing patient experience (PEx) in digital health. Neither the general PEx nor user experience (UX) adequately reflects the experience of a patient using a digital service. For example, in a hospital setting, the environment’s cleanliness, background noise, and even food provision could affect PEx [20]; however, these factors would not be expected to influence the experience of a patient using a digital service. Similarly, the fact that the system passes usability heuristics does not necessarily mean that the overall experience of a patient using digital health services is positive [21]. Therefore, it is vital to understand the experiences of individuals using digital health and how the design of new technologies can affect them [17,22,23].

The concept of (nondigital) PEx has many definitions in general health care practice and research. The Beryl Institute defines PEx as “the sum of all interactions, shaped by an organization’s culture, that influence patient perceptions, across the continuum of care” [24]. Other definitions and studies note that the core elements of optimized PEx include access to appropriate care, patients’ active participation in care, a good patient-physician relationship, reliable evidence-based care, comprehensible information, physical comfort, emotional support, involvement of family and friends, individualized approaches, responsiveness of services, and continuity of care [19,25-27]. These core elements of PEx help to recognize patients’ priorities when receiving care and in providing patient-centered care. However, patients’ priorities may differ for digital health, in which traditional face-to-face interaction is replaced by human to digital interface interaction. Therefore, to address patient priorities in digital health, it is essential to consider UX in the design of digital health [28]. In this study, we define UX as a person’s perceptions and responses that result from the use or anticipated use of a product, system, or service [18,29]. Usable, useful, findable, accessible, credible, valuable, and desirable products are more likely to succeed in delivering a positive UX [30]. However, the full impact of digital health technologies on PEx or UX still remains unclear [31]; some products even result in negative effects such as increased patient anxiety [32]. Therefore, more insights into the barriers to and facilitators of individuals’ experiences with digital health are required [33].

### Objectives

The objectives of this paper were to systematically review (1) the factors that influence PEx in digital health and (2) the design considerations of PEx that are in digital health. The overall aim was to generate a design framework and guidelines for further improving PEx in digital health.

## Methods

We performed an umbrella systematic review compiling evidence from multiple systematic reviews [34] on PEx and UX in digital health. This review was conducted according to the PRISMA (Preferred Reporting Items for Systematic Reviews and Meta-Analyses) methodology, which is an evidence-based minimum set of items for reporting in systematic reviews and meta-analyses [35].

### Digital PEx Working Definition

Throughout this study, we use the term digital PEx as a working definition to describe people’s experiences in various digital health contexts. As the study progressed, the definition underwent several revisions, which resulted in a more inclusive final definition.

### Search Strategy

We searched Scopus, PubMed, and Web of Science for studies published between January 1, 2000, and December 16, 2020. The search time window was limited to 2000 as the term digital health was first introduced by Frank [36] in 2000. To be inclusive, we used broad interchangeable search terms with varying combinations of digital health, PEx, and UX:

1. Category 1: “patient experience” OR “health experience” OR “user experience” OR “customer experience” OR “client experience”
2. Category 2: “ehealth” OR “e-health” OR “mhealth” OR “m-health” OR “telehealth” OR “tele-health” OR “digital health” OR “virtual health” OR “remote health” OR “telemedicine” OR “telemonitoring” OR “teleconsultation”
3. Category 3: “patient digital experience” OR “patient experience in digital health” OR “e-patient experience” OR “epatient experience” OR “online patient experience”

After combining categories 1, 2, and 3, limits were set to restrict studies to English-language literature reviews published in journals after 2000. The final search strategy was ([category 1 AND category 2] OR category 3) AND (DOCTYPE [review]) AND (PUBYEAR>2000) AND (LIMIT-TO [SRCTYPE, “journal”]) AND (LIMIT-TO [LANGUAGE, “English”]). Google Scholar was used as an additional database to manually search for additional related references based on the snowballing method during the review process.

### Selection Criteria

Eligibility criteria were developed for title and abstract screening and refined for full-text screening. The following inclusion criteria were proposed by TW and GG and adjusted by MM and RG:

1. No duplicated articles
2. Full text available
3. English language
4. Only completed peer-reviewed journal articles
5. Only review articles
6. Related to digital health (ie, use of information and communication technology in health) and PEx, UX, or health care experience

### Screening Process

The collected articles were included in the final analysis if they met all the inclusion criteria after a 2-stage screening process: first, a title and abstract review, followed by a full-text review. In the screening process, 2-round, small random samples (20%) were independently reviewed by 2 reviewers (TW and GG) who evaluated the eligibility of the articles against the selection criteria. The interrater reliability and clarity of the selection criteria were assessed using the Fleiss-Cohen coefficient until it reached the required strength (≥0.60). Uncertainties around paper inclusion and exclusion were resolved by discussions with the research team (TW, GG, MM, and RG) when necessary.

### Data Extraction and Thematic Analysis

Articles meeting the eligibility criteria were imported into ATLAS.ti (Scientific Software Development GmbH; version 9.0.7; 1857) for data extraction. Data were extracted for the following aspects: (1) study characteristics, including authors, year of publication, research aims, review methods, target users, and digital health intervention (DHI) characteristics; (2) the overall impression of digital PEx (eg, the foci or types of findings regarding digital PEx); (3) influencing factors of digital PEx; and (4) design considerations for improving digital PEx.

We used the Braun and Clarke 6-phase thematic analysis method [37] to analyze the extracted data; these include (1) familiarization with the data, (2) generation of initial codes, (3) searching for themes among codes, (4) reviewing themes, (5) defining and naming themes, and (6) producing the final report (analytical themes). A total of 4 researchers participated in the review process. After data familiarization, a set of a priori categories was defined by TW and refined by all the coauthors (Table 1). The coding was based on the Performance of Routine Information System Management (PRISM) framework [38], which states that routine HIS performance is affected by the system’s inputs (ie, technical, behavioral [39], and organizational determinants) and progress. Please note that other elements of the framework (outputs, outcomes, and impact) are discussed in another study addressing the evaluation of digital PEx (work in progress).

Group discussions among the authors were used to reach an agreement on the produced a priori categories. TW quoted the relevant data across the included reviews, generated initial codes based on a priori categories, and then searched for themes among codes. Frequently used terms in the included reviews were used as inspiration to generate subsequent codes and themes. The latter process was independently and randomly validated by GG, MM, and RG.

**Table 1:** A priori categories of influencing factors of digital patient experience based on the Performance of Routine Information System Management framework [38].

Determinants and a priori categories			Description
**Behavioral determinants**			
	Patient capability	The individual’s psychological and physical capacity to engage in the concerned digital health activity	
	Patient opportunity	The individual’s internal conditions that enable or disrupt patients to engage in digital health	
	Patient motivation	The reflective and automatic brain processes that energize and direct patients’ goal setting and decision-making and their behaviors regarding using digital health	
**Technical determinants**			
	Intervention technology	The integration of telecommunications and computers, as well as necessary enterprise software, middleware, and storage and audiovisual software, which enables users to access, store, transmit, understand, and manipulate health information	
	Intervention functionality	The ability of digital health to work as expected to help users meet their health goals and needs	
	Intervention interaction design	The process of moving digital health from its existing state to a preferred state to optimize interactions between patients and digital health interventions	
**Organizational determinants**			
	Organizational environment	The management of the health service system, as affected by the rules, values, and practices of the involved people or community	
	Physical environment	The tangible surroundings (such as space, light, or sound) around patients, which affects their interactions with digital health	
	Social environment	The cultural environment (such as policy, business, or customs) that affect patients’ interactions with digital health	

## Results

### Overview

**Figure 1:**
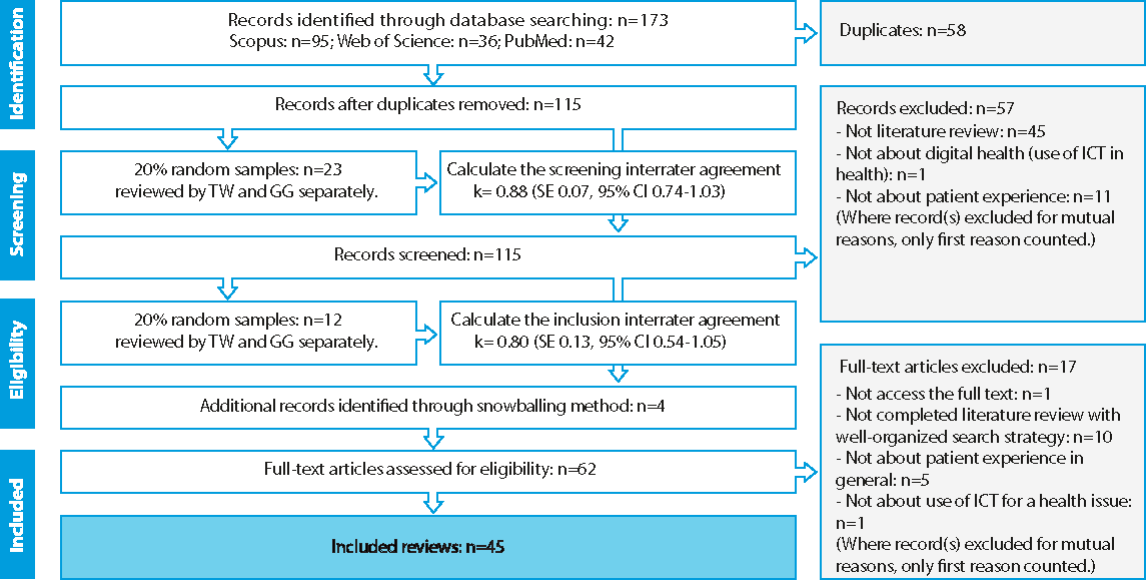
Study flow diagram. ICT: information and communications technology.

Figure 1 shows the flow diagram of the systematic search. A total of 173 records were generated after the computer search; 58 (33.5%) duplicates were removed, and the titles and abstracts of 115 (66.5%) articles were reviewed. Subsequently, 53.9% (62/115) of full-text articles (including 4 additional records collected through snowballing) were reviewed for inclusion. Ultimately, 45 studies were included in the review for data extraction.

### Study Characteristics

Embase, MEDLINE, PubMed, PsycINFO, CINAHL, and the Cochrane Library were the most common databases for the included reviews. Of these, 62% (28/45) were systematic review articles. The remainder included scoping reviews (6/45, 13%), literature reviews (3/45, 7%), integrative reviews (3/45, 7%), narrative reviews (2/45, 4%), comprehensive overviews (1/45, 2%), review of systematic reviews (1/45, 2%), and umbrella reviews (1/45, 2%). More than half of the included reviews (24/45, 53%) conducted quality assessments. The reviews included >1400 studies, which mainly or partially reported qualitative and quantitative analyses of PEx in digital health. The data analysis methods varied and included thematic analysis (8/45, 18%), meta-synthesis (5/45, 11%), meta-ethnography synthesis (2/45, 4%), taxonomy (1/45, 2%), hermeneutic synthesis (1/45, 2%), qualitative evidence synthesis (1/45, 2%), and state-of-the-art survey analysis (1/45, 2%).

Among the included reviews, some focused on specific populations, such as children (3/45, 7%), college students (1/45, 2%), younger people (1/45, 2%), adults (7/45, 16%), or older adults (4/45, 9%). Others either focused on the general population or did not mention the target population. The most common health issues across the included articles were chronic diseases (17/45, 38%), including chronic obstructive pulmonary disease, heart failure, cardiovascular disease, cancer, diabetes, and hypertension. Mental health problems (7/45, 16%), including depression, anxiety, psychological well-being, psychotic disorders, and schizophrenia, were the second most common health issues. The remainder either focused on other issues (8/45, 18%), including audiology, asthma, reproductive health, maternal health, newborn health, child health, adolescent health, surgery, postpartum, somatic diseases, or palliative care, or did not mention any specific health issues (14/45, 31%). Some papers (8/45, 18%) also provided multistakeholder perspectives, including health care professionals, providers, surgeons, clinicians, staff and organizations, implementers (such as health policy makers, clinicians, and researchers), and the participation of information technology.

The degree of detail provided about the interventions varied greatly across the studies. Phone-based apps, websites, handheld sensing devices, and ambient assisted living health care systems were common digital health deliveries. Interaction techniques included synchronous, asynchronous, and hybrid models. Diverse intervention platforms, systems, or functions were used to deliver various health care services, including supporting disease management (14/45, 31%); patient-to-physician communication or consultation (9/45, 20%); symptom monitoring (9/45, 20%); information transmission (4/45, 9%); health promotion activities (3/45, 7%); screening, diagnosis, or self-assessment (2/45, 4%); behavior changes (2/45, 4%); self-education (1/45, 2%); and decision-making (1/45, 2%). Multimedia Appendix 1 [28,40-83] provides detailed information regarding the characteristics of the included studies.

### Overall Impression of Digital PEx

Our study revealed great diversity in the perspectives and definitions describing patients’ experiences and characteristics when using digital health, presenting a variety of influencing factors and design considerations for digital PEx. The included studies showed different foci regarding digital PEx, including influencing factors (21/45, 47%) [28,40-59], digital health performance (19/45, 42%) [40-43,46,48,49,56,57,59-68], patient perceptions (9/45, 20%) [28,45,47,49,69-73], evaluation methods of digital health or digital PEx (8/45, 18%) [43,64,74-79], and design considerations (9/45, 20%) [48-50,53,54,59,80-82]. The findings and conclusions of the 45 reviews showed a great diversity. Most studies presented a set of themes (19/45, 42%) [28,44,45,48,49,51,54-57,59,62,69-73,76,79] or descriptions only (16/45, 36%) [40-42,46,47,58,60,61,63-67,74,75,83]. Other studies concluded with a theory-based description (5/45, 11%) [52,68,77,78,80], framework (4/45, 9%) [28,49,50,82], model (2/45, 4%) [53,69], method (2/45, 4%) [43,81], or checklist (1/45, 2%) [59]. Only a few studies transformed findings into design considerations (9/45, 20%) or visualized or structured their results into frameworks, models, checklists, or methods (9/45, 20%). Limited information was found on participant dropout reasons during the interventions [28,41,43,51,53,63,69,71]. The overall impression of the researchers on the DHIs was positive. In 51% (23/45) of reviews [41-44,48,49,52-54,57,59,61-63,65-69,72,73,77,80], the DHIs either showed promising results or at least results comparable with face-to-face health care services. Only 4% (2/45) of reviews [47,60] reported concrete evidence of the negative impact of current DHIs on digital PEx. In general, digital PEx was addressed because of the interactions between the DHIs and the patients involved and how the service was organized and carried out.

### Influencing Factors of Digital PEx

An influencing factor is an aspect of the existing situation that influences other aspects of the situation, and it is formulated as an attribute of an element that is considered relevant and can be observed, measured, or assessed [84]. In this study, influencing factors refer to specific factors that lead to a positive or negative experience (digital PEx). Some factors have either positive or negative consistent and concrete impacts, whereas others have double-edged impacts; that is, impacts that are different per individual or change over time. Among the included papers, a common understanding of the potential influencing factors was captured from 3 aspects—behavioral, technical, and organizational determinants—following the categorization of the PRISM framework. These determinants were each classified into 3 categories, resulting in nine categories: patient capability, patient opportunity, patient motivation, intervention technology, intervention functionality, intervention interaction design, organizational environment, physical environment, and social environment. Multimedia Appendix 2 [28,40-83] presents an overview of the themes identified for each category, the influencing factors per theme (positive, negative, and double-edged), and references. Most factors appear to be related to technical determinants, followed by behavioral and organizational determinants. For *technical determinants*, we summarized 3 categories with 13 themes, containing 58 positive, 35 negative, and 13 double-edged factors. For example, DHIs with multiple behavioral change techniques appeared to be more effective [42,56,80] and reported higher patient satisfaction [54,57]. *Behavioral determinants* included 3 categories with 9 themes containing 11 positive, 21 negative, and 5 double-edged factors. For instance, some studies mentioned a lack of confidence in patients’ own ability to use the technology [43,45,47,48,71,81], leading to a negative digital PEx. *Organizational determinants* were classified into 3 categories with 5 themes, including 13 positive and 23 negative factors. For example, unrealistic financial reimbursement and higher costs related to the internet or equipment were practical challenges of using digital health [47,48,51,55,56]. For the behavioral and organizational determinants, we collected more negative factors than positive factors. This is in contrast to the technical determinants, in which more positive factors were identified. Double-edged factors were less than both positive and negative factors for all the 3 determinants. Multimedia Appendix 3 [28,40-61,63-67,69-73,75-78,80-83] provides detailed information and examples.

### Design Considerations of Digital PEx

Table 2 provides an overview of the identified themes for each design construct or method, related considerations, and references. To address the abovementioned influencing factors, several the included articles referred to *design constructs* (personalization, information, navigation, and visualization) [48,49,53,54,59,80] and *design methods* (ie, human-centered design [HCD] or user-centered design [UCD], co-design or participatory design, and inclusive design) [48-50,54,80-82], either as recommendations or implications for improving digital PEx from a design perspective. Notably, there was an overlap between design considerations and influencing factors. The former focuses on concluding possible design suggestions, recommendations, and implications proposed by the reviewed articles. The latter involves mapping the impacts of interaction design on digital PEx in different contexts; therefore, they refer to different themes and references. Generally, the *personalization construct* identifies patient profiles and tailors digital health according to patients’ needs and preferences. The *information construct* addresses the source, language, presentation, content, and architecture of delivered health information. The *navigation construct* considers the interactive, delivered, and instructional elements of digital health to guide users to different areas of content within digital health. The *visualization construct* focuses on the aesthetics, attractiveness, visibility, and consistency of digital health appearance and interface. Furthermore, co-design and UCD or HCD were recommended as the most common methods for designing digital health, which involve multi-stakeholders and multi-disciplinaries in the design process to facilitate the designers’ work, as designers need to understand end user needs and be aware of potential barriers to engaging in DHIs. Finally, *inclusive design* provides flexible design and is usable for a broader population. Notably, the design considerations identified in the included papers are not meant to be applied to every project; the implementation depends on the project’s focus. Designers always need to balance project requirements (such as profits), user needs (such as privacy concerns), and policy regulations (such as data security). For example, peer-to-peer patient communication may not be appropriate for more sensitive health issues.

**Table 2:** Design considerations of digital patient experience.

Themes				Considerations		References
**Design constructs**						
	**Personalization**					
		Profiling	Careful patient selection for digital health use Assess specific metrics (eg, sociodemographic characteristics, basic health status, individual preferences, and habits) Create an accurate patient profile		[53,59]	
		Tailoring	Provide personalized information, tailored intervention content, and customized feedback (eg, predicted possible causes and consequences of a health problem and advice on the behavior under investigation) Tailor the content to the user’s needs and preferences Tailor images, colors, text quantity, and font size and color to what users find appealing Tailor multiple variables rather than a single variable Align with end users’ habitual routines		[49,54,59,80]	
		Autonomy	Choose desirable and accessible forms of delivery Choose when and how to receive reminders Select or change personalized goals for future use throughout the time span of intervention Select preferred styles (eg, color and font)		[59,80]	
	**Information**					
		Content	Provide comprehensive health information (eg, medical history, test results, and medication information) Provide appropriate education and training on a health condition Provide concise information (not overwhelm) Provide evidence-based information from a credible source (eg, no advertisements and validated advice) Appropriate encryption and digital health security (eg, password setting and privacy policy)		[48,53,54,59]	
		Communication	Provide peer-to-peer communication through web-based forums and communities using instant messages Access to professionals directly via email, SMS text message, or live chat Share duties between health care staff		[53,54,59,80]	
		Functionality	Rewards (eg, material incentives, intangible rewards, and messages of congratulations when a task is completed) Reminders (eg, email messages, SMS text messages, words of the day, and pop-ups) for task completion Reflective feedback, persuasive features, and gaming features (eg, knowledge quizzes and games) Functional characteristics enable accurate and continuous self-management (eg, activity planning, activity tracking, self-monitoring, and diaries), person-centered care, and sustained behavior change Appropriate dose of treatment		[48,49,53,54,59,80]	
	**Navigation**					
		Forms of delivery	Readily accessible and downloadable Improve DHI^a^ delivery (eg, change from a website to a mobile phone app) Ability to print and email the information		[49,54]	
		User flows	Minimum input (eg, voice commands) Efficient access to information Clarify what to do next Provide search bar and menu bar Tools and aids to help understand health information and complete health tasks		[48,59]	
		Instruction and tutorials	Guide users to a greater extent if the design is not self-explanatory Provide more concrete, explicit, and context-sensitive instructions (eg, a virtual guided tour and extra internal links) Adopt features from common (ie, familiarized) user interfaces (eg, the iPhone interface) Provide appropriate education and training on digital health use		[54,80]	
	**Visualization**					
		Message presentation	Visualize continuous monitoring data (eg, present data as graphs and tables) Provide a coherent presentation in terms of colors, pictures, and themes Simple nontechnical language Straightforward and concise text Comprehensive descriptions of actionable message Provide positive, nonauthoritarian, friendly, and nonjudgmental tone of voice Multimedia messages (eg, text combined with relevant pictures or video) Highlight information using various font styles, sizes, and colors		[59]	
		Interface aesthetic	Show graphics (ie, visual aids) rather than (too much) text Provide a pleasing color scheme (eg, bright colors) Simple interface		[54,59,80]	
**Design methods**						
	**Co-design and participatory design approaches**					
		Multistakeholder	Involve end users and other stakeholders Include the user at the beginning of the design process		[48,49,54]	
		Interdisciplinary	An interdisciplinary approach to the development and implementation		[48,80,81]	
	**User-centered design and human-centered design approaches**					
		Needs assessment	Know the needs, capabilities, and environment of users through focus groups, surveys, interviews, and personas Composing, preparing, and organizing content		[48,81,82]	
		Usability testing	Gain early feedback from users through prototypes; benchmark testing, user testing, heuristic analysis, failure modes and effects analysis, and observations in other health care settings		[48,81,82]	
		Implementation	Fit the technology to the person, not the person to the technology; pilot testing, task analysis, and reporting mechanism		[81,82]	
		Monitor and sustain	Understanding work as imagined often differs from work as done; pre- and posttesting, contextual inquiry, and safety and hazard reporting		[82]	
	**Inclusive design approaches**					
		Inclusive	Provide a flexible design that is usable by people with no limitations, as well as by people with functional limitations related to disabilities or old age		[48,50]	

^a^DHI: digital health intervention.

### Design Implications

On the basis of our findings regarding influencing factors and design considerations for digital PEx, in this section, we define digital PEx and present design guidelines for the implementation of improving PEx in digital services.

#### Definition of Digital PEx

Our review reveals the absence of a commonly used concept for PEx in digital health. An increasing number of studies have been conducted on surveying PEx, satisfaction with, and expectations in varied digital health. With the growing academic interest in this topic and increasing efforts to address PEx in digital health design practice, a common concept with a concise definition will strengthen and align efforts overall. After reviewing the alignment of widely accepted concepts of PEx, UX, and DHIs with our generated influencing factors, we observed that many of our findings are included in the PEx definition offered by The Beryl Institute. Therefore, by including the sum of all interactions shaped by an organization’s culture, which influence patient perceptions across the continuum of care [33] along with the constructs of UX (people’s perceptions and responses [18,29]), DHIs (digital health technologies [13]), and the determinants (ie, technical, behavioral, and organizational determinants) identified in this review, we propose a concise, practical definition of digital PEx to guide the future design of digital health: “Digital patient experience is the sum of all interactions, affected by a patient’s behavioral determinants, framed by digital technologies, and shaped by organizational culture, that influence patient perceptions across the continuum of care channeling digital health.” Compared with the original definition of general PEx, this new definition underlines the digital part of health care delivery and includes 2 new determinants (technical and behavioral) that go beyond the organization’s culture to clarify what can influence patient perceptions while traveling along a digital care pathway.

#### Design Guidelines for Improving Digital PEx

**Figure 2:**
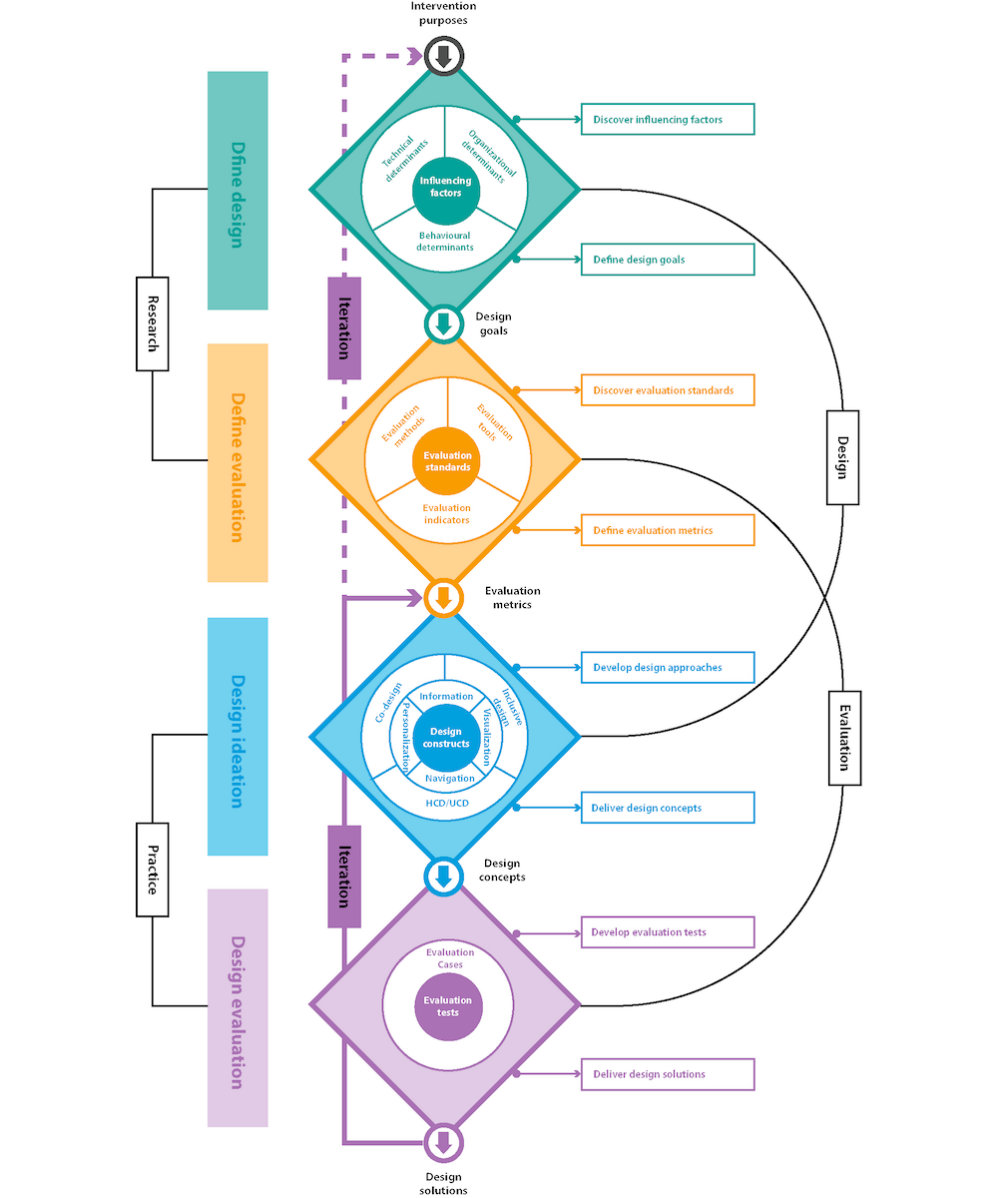
Digital patient experience design and evaluation framework. HCD: human-centered design; UCD: user-centered design.

We developed a design and evaluation framework to help digital health designers or developers improve digital PEx in the design process (Figure 2). This framework was based on the findings of this umbrella review and was inspired by the double diamond model [85,86]. Our framework shows four phases: *define design, define evaluation, design ideation,* and *design* *evaluation*. The first and third phases focus on the design itself, and the second and fourth phases focus on design evaluation. In this study, we focus on explaining the first and third phases. In the first phase, designers must define the design goals by considering the factors that affect digital PEx. In this phase, we provided 3 determinants referring to 9 categories of influencing factors that have 3 types of impact on digital PEx (positive, negative, and double-edged) for designers to discover and explore. Designers can frame their design goals based on the intervention purposes and the selection of influencing factors. For example, if the purpose of the intervention is to improve patient eHealth literacy, designers need to pay more attention to patient capability and frame a design goal to develop suitable intervention functionality for improving patient capability. After defining the design goals, designers can move to the second phase, which is the *define evaluation* phase. In this phase, designers need to consider evaluation indicators (patient emotional, behavioral, and health outcomes) and evaluation methods (surveys and interviews) that are used to assess digital PEx. Detailed information regarding this phase will be discussed in a parallel study. Following this, we provide 4 design constructs (personalization, information, navigation, and visualization) and 3 design methods (ie, HCD or UCD, co-design, and inclusive design) for the design ideation phase. *Personalization* [41,54,56,57,59,69,81,87] refers to ascertaining user needs with design goals. It encompasses the design of intervention technology and functionality needs that meet the patients’ ability, opportunity, and motivation to trigger behavior changes and promote health outcomes. *UCD/HCD* and *inclusive design* are valuable at this stage for the inclusion of patient perspectives. Driven by user needs and intervention goals, *information* includes content, communication, and functionality [54,59,81], and *navigation* comprises forms of delivery, user flows, instructions, and tutorials [54,59,80,81]. This relates to how relevant content presented in multimedia with a clear information architecture can attract patient attention and help them understand and complete tasks efficiently [88]. *Co-design* and *participatory design* are multidisciplinary collaborations that are necessary at these 2 stages. Finally, designers need to consider *visualization* [54,57,59,80,81], which determines the product look. The digital health interface can affect patients’ first impressions when using DHIs. An attention-grabbing, simple, and consistent interface [59], layout (colors and images) [80], and message presentation [59] can all lead to positive UX. The design guidelines (Textbox 1) can be used at this stage to produce design concepts. In addition, this phase contains the digital health design workflow, challenges, and tips from a design practice perspective (which will be presented in an ongoing interview study). Finally, we ended up with this framework by introducing the design evaluation phase, in which designers need to develop tests (based on evaluation metrics) to evaluate design concepts. If the evaluation outcomes do not meet the evaluation standards, designers can return to the design ideation phase to adjust the design concepts or return to the first phase to reconsider the design goals.

Compared with the original double diamond model, our framework separates the evaluation part from the design part. This aligns with the design research methodology framework [84], which suggests generating success criteria after clarifying design research goals and before producing design support, formulating criteria for success is essential to be able to determine whether the results help achieve this aim. Therefore, we paid equal attention to design and evaluation. In addition, our framework provides detailed reference materials (such as 3 determinants) for each phase to provide designers with more practical support. Notably, in our framework, we retain some typical features of the double diamond model: the first 2 phases are research related, the last 2 phases are practice related, and each phase starts from divergence and ends at convergence.

On the basis of our findings on influencing factors and design considerations, we mapped the combinations of design constructs and design methods into 9 design guidelines to address different influencing factors (Textbox 1), which can be used to guide the design ideation process. Some of the design guidelines uncovered in this study have already been implemented, resulting in a positive digital PEx, such as the digital platform PatientsLikeMe, which aims to empower patients to navigate their health journeys together through peer support, personalized health insights, tailored digital health services, and patient-friendly clinical education [89]. One of the studies pointed out that patients can greatly benefit from using this platform as it improves patient health literacy, and its condition-specific customization may still further improve PEx [90], which aligns with our design guidelines on improving “patient capability” and providing “personalized information.”

**Design guidelines for improving digital patient experience.**

**Categories and design guidelines**

- Patient capability
  Identify patients’ knowledge and skill levels by understanding their technology, language, and health literacy; consider their previous experience and current confidence level in using digital health; improve their actual literacy and correct their perceived inability; tailor design to their ability
- Patient opportunity
  Profile patients’ identity (eg, age, gender, economic status, and daily routines) and health status (eg, illness complexity, severity, and stability); consider patients’ accessibility and affordance to digital health; tailor design to their individual opportunity
- Patient motivation
  Recognize patients’ mindset and perceived advantages and disadvantages; inform them of the potential benefits of using digital health; address their concerns and worries; understand their expectations and needs; tailor design to their preferences to trigger their motivation
- Intervention technology
  Increase technical usability; ensure ease of use, ready to use, and timely feedback on digital health; select technical features (eg, data accessibility) and delivery media or devices (eg, device ownership) to meet patients’ preferences and needs
- Intervention functionality
  Strengthen theory-based interventions (eg, behavior change techniques and evidence-based interventions); improve intervention quality, considering privacy, security, and accuracy issues; provide regular and continuous social support combining both remote communication and real human contact; tailor health promotion and intervention structure to patients’ needs and preferences
- Intervention interaction design
  Provide personalized and consistent information, clear tutorials or technical support, and visualized data; allow patients to choose personalized interactive elements; follow human or user-centered design, co-design, and inclusive design methods; involve multi-stakeholders and multi-disciplines in the design process
- Organizational environment
  Reduce equipment or service cost and time; improve health care providers’ professional ability, communication skills, and service attitudes across the use of digital health; increase workflow transparency and clarify accountability; improve system integration and compatibility
- Physical environment
  Provide a familiar, warm, and comfortable environment rather than cold and unfamiliar settings; reduce environmental distractions (eg, background noise or lighting)
- Social environment
  Provide adequate support policies and legislation; develop plausible business cases

## Discussion

### Principal Findings

We systematically reviewed review articles on factors that influence digital PEx and considerations regarding how best to design digital PEx. The reviews varied greatly in type, including studies and data analysis methods, as well as in HIS, health issues, target patient groups, intervention content, and structure. Of the selected reviews, 62% (28/45) were systematic reviews, the rest were *other* types. These included qualitative, quantitative, and mixed methods studies. Thematic analysis and meta-analysis were the most common data analysis methods used in the reviews. We note that the studies described in the selected reviews were extremely heterogeneous, and information about interventions and digital PEx were often mixed and complex, making comparison difficult.

Our results are in line with the findings reported by previous authors [25,30,38] on the factors that affect PEx, UX, or the implementation of digital health. On the basis of the identified influencing factors and design considerations, we developed 9 design guidelines for improving digital PEx. Our findings reveal that among the selected reviews, only a few formulated design strategies or guidelines. This lack of design knowledge transformation makes it difficult for designers or developers to apply the findings directly. This aligns with the studies by Sakaguchi-Tang et al [48] and Søgaard Neilsen and Wilson [80]; the former indicated that the absence of specific design recommendations impairs the design of digital health, with the latter suggesting that there was a lack of understanding of the most beneficial design aspects for some specific digital health and how design principles can best be applied. Moreover, the use of UCD has been recommended in many studies to address UX-relevant issues in digital health [3,80,91], which also supports our findings.

### Digital PEx Versus General PEx and UX

We found a lack of a common term to describe PEx in digital health; UX (25/45, 56%) and PEx (17/45, 38%) were the most commonly used terms. Patient UX, patient perceptions, client experiences, patient empowerment, and user engagement were also used to describe similar concepts. Many reviews indicated that there was limited information about UX or PEx in varied digital health and underlined the need for a more holistic view of patient needs and priorities to better shape digital health design strategies and provide tailored digital health [28,40,42,45,60,74].

### Influencing Factors Are More Complex Than Facilitators and Barriers

The information provided about digital PEx–influencing factors was complex and heterogeneous. Digital health is often treated as a whole, whereas digital PEx is affected by the additive effect of varying digital health factors. A single change in a factor may affect everything else. We found that without a concrete interaction context, factors could be regarded concurrently with facilitators or barriers. For example, regular contact with health care providers (HCPs) could be perceived to increase a sense of reassurance or perceived as a burden to patients’ daily lives [28]; some patients experienced digital health as time consuming or an additional burden, whereas others experienced it as time saving or convenient [69]. Some influencing factors may have a soft or indirect influence on digital PEx [44,76]. For instance, users who are completely unaware of privacy or security risks may have excellent experience with digital health that fails to meet privacy or security requirements [76]. A lack of concrete solutions to address these barriers was mentioned [48]. It is likely that digital health cannot serve all populations equally well [71], which aligns with the results of a scoping review that investigated the inequities caused by the adoption of digital health technologies [92]. Some researchers indicated that older adults can also experience benefits by using digital health [53], whereas others suggested that telehealth is, at best, a partial solution for younger and fitter subpopulations [47,71]. Again, although some mentioned that patients preferred using personal devices [49,55,61,64], others noted the opposite [64].

### Unclear Benefit From the Different Elements in Digital Health

It is likely that some patient groups benefit more than others from specific DHIs. For example, one of the reviews suggested that in telemedicine treatment for type 2 diabetes, behavioral change and continuous management were the keys to success [43]. However, it was unclear precisely which elements of digital health resulted in patients’ satisfaction or dissatisfaction and how they could be addressed [83]. Moreover, we found limited data and even contradictory results on which factors affect digital PEx the most, which elements should be considered first when developing DHIs, and who benefits more from them. The latter is commonly mentioned [48,53,70], with some authors suggesting that patients with unstable chronic diseases might benefit the most [47,93]. However, another review indicated that even if patients are provided with the latest state-of-the-art technology at home, the intervention will not be beneficial if it remains unused [43]. Patients who are less activated are likely to have less positive experiences than those who are highly engaged [74].

### Lack of Multiple Perspectives During the Design of Digital PEx

Clear communication between experts, designers, and patients regarding their understanding of digital PEx is required. Some reviews acknowledged the need for a multistakeholder perspective on digital PEx [55,69]. However, we found circumstances in which this was not possible. For example, in some cases, UCD for DHIs was conducted on nonpatient users either because of ethical reasons or relevant regulations [43], and in others, apps that are not specifically designed for patients with cancer were being used for this patient group [64]. HCPs are often isolated from the decision-making process to incorporate digital health into their current service provision [28]. Moreover, a lack of clinician perceptions of digital health use was also reported [40,54]. Furthermore, no studies focused on exploring designers’ views, opinions, experiences, or values in addressing PEx or UX in the design of digital health. There was little information on whether experienced designers had worked with patients in their design process.

### Over- or Underestimated Results

Some studies suggested that a lack of interest was the main reason for patients’ refusal of digital health and that reasons for patient withdrawal were patients not wanting to use equipment, deteriorating health, and technical problems [94,95]. We need to gain better insights into the reasons for patients choosing not to engage in or withdraw from digital health, as these will significantly inform future DHI development and design [43,53,69]. However, it is likely that most studies only included patients who had already agreed to or were using digital health technologies; those who refused to use, withdrew from, or had no accessibility were excluded [28,51,63,69]. One of the reviews suggested that this would result in over- or underestimated results of DHIs’ effects on digital PEx, as participants who completed the intervention may differ from those who did not [41]. Another review found that patients only reported positive themes associated with remote monitoring, which may indicate a selection bias [71].

### Conflicts Between Benefits and Cost for Developing DHIs

The provision of digital health can reduce the treatment burden and better integrate care into patients’ daily routines [69], which is consistent with our findings; we found that most reviews had a positive perspective of DHIs. However, in one of the reviews, it was suggested that although there was agreement among most professionals that health information technology can have a positive impact on PEx, when weighing the benefits against the potential cost, demonstrating this will be challenging [44,47]. Moreover, unnecessary high-frequency monitoring could result in a waste of health resources and an increased workload for HCPs [52]. Compared with existing health care services, the application of new technology needs to demonstrate clinical evidence of improved health conditions [43]. However, there were discordant findings in terms of the benefits of using DHIs. For example, there was no concrete evidence that telemedicine consultations were quicker than face-to-face consultations [40,57,68,83]. In another case, the impact of DHIs on health care use was not examined [57]. In conclusion, only user-friendly and quality-certified DHIs should be provided to patients [64]; health care organizations should not shift their focus from the basic and inexpensive strategies that affect patient care. Care is needed: new technology should not overwhelm the patient or ignore patient needs [44].

### Limitations

First, when undertaking a review of review articles, some important details included in the original studies may have been lost, which increases the possibility of reporting bias. We also noted differences in the interpretation of terms and methods between the reviews. There is a lack of consistency in the terminology used to describe the functions of DHIs, HISs, or digital PEx itself. For example, in some cases, “eHealth” and “mHealth” were used as interchangeable terms [75], “persuasive technology” and “behavior change techniques” were presented as having a similar meaning [43], and “patient engagement” and “patient activation” were also regarded as being the same [74]. This inconsistent use of terms may impede knowledge translation and dissemination [57]. To counter this, we summarized the varied factors with unified descriptions to build a common understanding of the digital PEx–influencing factors.

Second, the intervention types and patient groups varied widely among the reviews, limiting meaningful comparisons between different studies. In addition, the digital health landscape is rapidly evolving, and the technology infrastructure is constantly shifting [41], as are the continuous updates of the UX design area. It is important to keep the influencing factors updated or adapted as the technology develops. Possibly, relevant original studies may have been excluded because of our focus on review papers. However, our approach to conducting an overarching review provides readers with a quick overview of the relevant digital PEx studies and a basis for further research.

Third, our umbrella review did not account for the multimodal relationships between subthemes or the potential overlap between subthemes within different domains. For example, different subthemes, such as “personalized design” in “interventions’ interaction design” also interconnect with “interventions’ technology” and “interventions’ functionality.” Moreover, our review process did not aim to address the question of whether some influencing factors are more important than others or how different aspects of DHIs influence them. This warrants further investigation as we suspect that differences may exist between the influencing factors, as some elements in digital health are more likely to increase or inhibit a positive digital PEx.

Finally, as we used qualitative thematic analysis to synthesize the findings and generate themes, the generated themes could have been influenced by the authors’ previous research experiences and personal understanding. By asking other researchers to repeat the coding process, the resulting themes are likely to be different. However, to minimize the potential coding bias, the generation of categories was based on the PRISM framework; 4 researchers with different backgrounds, including design, medical, and human factors, were involved in the iterative coding process, group discussion, and independent and random validation, and existing theories were used.

### Further Research

The goals of this umbrella review were to systematically review the influencing factors that affect digital PEx and the design considerations for improving digital PEx that are summarized in the existing literature. We must conclude that, currently, much remains unknown, and the topic of digital PEx is relatively new. We propose 6 directions that require further research. The first direction is to develop frameworks or models that translate digital PEx–related research findings into design practices or implications. For example, in this study, we used design guidelines and a design framework to summarize the findings. The second direction is to identify those who will benefit more from which elements in DHIs and which influencing factors could be addressed by combining design constructs and design methods. The third direction is to further examine how designers understand and address digital PEx in the digital health design process. To address this, we conducted a qualitative study on how designers address digital PEx in design practice. The fourth direction is to standardize evaluation indicators, methods, or tools for assessing digital PEx; we are currently evaluating digital PEx in a parallel study. The fifth direction is to quantify the balance between the benefits and costs of developing user-friendly and validated DHIs. The sixth direction is to identify participants’ reasons for dropping out and their impact on the reported digital PEx–related results.

### Conclusions

To the best of our knowledge, this is the first study to propose the term “digital patient experience” as a common phrase to describe PEx in digital health and define digital PEx by synthesizing the reported PEx or UX of varied DHIs from multiple reviews. Multimedia Appendix 4 shows more details about the structure of this study. In this review, information on influencing factors was identified and summarized into 9 categories (ie, patient capability, opportunity, motivation, intervention technology, functionality, interaction design, organizational, physical environment, and social environment). These categories were classified into positive, negative, and double-edged factors based on their positive, negative, and diverse impacts on digital PEx. Our review uncovered 4 design constructs (personalized, information, navigation, and visual design) and 3 common design methods (UCD or HCD, co-design, and inclusive design) as design considerations for addressing digital PEx. Finally, we proposed a design and evaluation framework and design guidelines to help digital health designers and developers address digital PEx throughout the entire design process.

## Appendix

Multimedia Appendix 1 Study characteristics and digital health intervention characteristics of included reviews.

Multimedia Appendix 2 Influencing factors on digital patient experience (double-edged factors imply diverse impact, positive factors imply positive impact, and negative factors imply negative impact).

Multimedia Appendix 3 Detailed information on themes of influencing factors of the digital patient experience.

Multimedia Appendix 4 PRISMA (Preferred Reporting Items for Systematic Reviews and Meta-Analyses) checklist.

## Abbreviations

DHI: digital health intervention
HCD: human-centered design
HCP: health care provider
HIS: health information system
PEx: patient experience
PRISM: Performance of Routine Information System Management
PRISMA: Preferred Reporting Items for Systematic Reviews and Meta-Analyses
UCD: user-centered design
UX: user experience

## References

[1] (2021). Second round of the national pulse survey on continuity of essential health services during the COVID-19 pandemic. World Health Organization.

[2] Mehrotra A, Ray K, Brockmeyer DM, Barnett ML, Bender JA (2020). Rapidly converting to “Virtual Practices”: outpatient care in the era of Covid-19. NEJM Catalyst Innovations in Care Delivery.

[3] Marcin JP, Shaikh U, Steinhorn RH (2016). Addressing health disparities in rural communities using telehealth. Pediatr Res.

[4] (2016). Atlas of eHealth country profiles: the use of eHealth in support of universal health coverage. World Health Organization.

[5] Davis SW, Oakley-Girvan I (2015). mHealth education applications along the cancer continuum. J Cancer Educ.

[6] Bender JL, Yue RY, To MJ, Deacken L, Jadad AR (2013). A lot of action, but not in the right direction: systematic review and content analysis of smartphone applications for the prevention, detection, and management of cancer. J Med Internet Res.

[7] Arnberg FK, Linton SJ, Hultcrantz M, Heintz E, Jonsson U (2014). Internet-delivered psychological treatments for mood and anxiety disorders: a systematic review of their efficacy, safety, and cost-effectiveness. PLoS One.

[8] Pal K, Dack C, Ross J, Michie S, May C, Stevenson F, Farmer A, Yardley L, Barnard M, Murray E (2018). Digital health interventions for adults with type 2 diabetes: qualitative study of patient perspectives on diabetes self-management education and support. J Med Internet Res.

[9] McLean G, Band R, Saunderson K, Hanlon P, Murray E, Little P, McManus RJ, Yardley L, Mair FS, DIPSS co-investigators (2016). Digital interventions to promote self-management in adults with hypertension systematic review and meta-analysis. J Hypertens.

[10] Escriva Boulley G, Leroy T, Bernetière C, Paquienseguy F, Desfriches-Doria O, Préau M (2018). Digital health interventions to help living with cancer: a systematic review of participants' engagement and psychosocial effects. Psychooncology.

[11] Augenstein J (2020). Opportunities to expand telehealth use amid the coronavirus pandemic. Health Affairs.

[12] Tecco H (2017). 2016 Year end funding report: a reality check for digital health. Rock Health.

[13] (2020). Digital implementation investment guide (DIIG): integrating digital interventions into health programmes. World Health Organization.

[14] Irizarry T, DeVito Dabbs A, Curran CR (2015). Patient portals and patient engagement: a state of the science review. J Med Internet Res.

[15] Free C, Phillips G, Galli L, Watson L, Felix L, Edwards P, Patel V, Haines A (2013). The effectiveness of mobile-health technology-based health behaviour change or disease management interventions for health care consumers: a systematic review. PLoS Med.

[16] Ammenwerth E, Schnell-Inderst P, Hoerbst A (2012). The impact of electronic patient portals on patient care: a systematic review of controlled trials. J Med Internet Res.

[17] Alkire (née Nasr) L, O'Connor GE, Myrden S, Köcher S (2020). Patient experience in the digital age: an investigation into the effect of generational cohorts. J Retail Consum Serv.

[18] Bolton RN, McColl-Kennedy JR, Cheung L, Gallan A, Orsingher C, Witell L, Zaki M (2018). Customer experience challenges: bringing together digital, physical and social realms. J Serv Manag.

[19] Kneeland PP, Anderson ME, Glasheen JJ (2016). Patient experience. Hospital Medicine Clinics.

[20] Reeves R, Coulter A, Jenkinson CJ, Cartwright J, Bruster S, Richards N (2002). Development and pilot testing of questionnaires for use in the acute NHS trust inpatient survey programme. Europe: Picker Institute.

[21] Richardson B, Campbell-Yeo M, Smit M (2021). Mobile application user experience checklist: a tool to assess attention to core UX principles. Int J Hum Comput Interact.

[22] Kellermann AL, Jones SS (2013). What it will take to achieve the as-yet-unfulfilled promises of health information technology. Health Aff (Millwood).

[23] Larivière B, Bowen D, Andreassen TW, Kunz W, Sirianni NJ, Voss C, Wünderlich NV, De Keyser A (2017). “Service Encounter 2.0”: an investigation into the roles of technology, employees and customers. J Bus Res.

[24] Defining Patient Experience. The Beryl Institute.

[25] Staniszewska S, Boardman F, Gunn L, Roberts J, Clay D, Seers K, Brett J, Avital L, Bullock I, O' Flynn N (2014). The Warwick Patient Experiences Framework: patient-based evidence in clinical guidelines. Int J Qual Health Care.

[26] (2011). NHS Patient Experience Framework. Department of Health, National Health Service.

[27] Shandley LM, Hipp HS, Anderson-Bialis J, Anderson-Bialis D, Boulet SL, McKenzie LJ, Kawwass JF (2020). Patient-centered care: factors associated with reporting a positive experience at United States fertility clinics. Fertil Steril.

[28] Brunton L, Bower P, Sanders C (2015). The contradictions of telehealth user experience in chronic obstructive pulmonary disease (COPD): a qualitative meta-synthesis. PLoS One.

[29] Jokela T, Iivari N, Matero J, Karukka M (2003). The standard of user-centered design and the standard definition of usability: analyzing ISO 13407 against ISO 9241-11. Proceedings of the Latin American conference on Human-computer interaction.

[30] Morville P (2005). Experience design unplugged. ACM SIGGRAPH 2005 Web program.

[31] Mobasheri MH, Johnston M, King D, Leff D, Thiruchelvam P, Darzi A (2014). Smartphone breast applications - what's the evidence?. Breast.

[32] Foley NM, O'Connell EP, Lehane EA, Livingstone V, Maher B, Kaimkhani S, Cil T, Relihan N, Bennett MW, Redmond HP, Corrigan MA (2016). PATI: patient accessed tailored information: a pilot study to evaluate the effect on preoperative breast cancer patients of information delivered via a mobile application. Breast.

[33] Wolf JA, Niederhauser V, Marshburn D, LaVela SL (2014). Defining patient experience. Patient Exp J.

[34] Grant MJ, Booth A (2009). A typology of reviews: an analysis of 14 review types and associated methodologies. Health Info Libr J.

[35] Liberati A, Altman DG, Tetzlaff J, Mulrow C, Gøtzsche PC, Ioannidis JP, Clarke M, Devereaux PJ, Kleijnen J, Moher D (2009). The PRISMA statement for reporting systematic reviews and meta-analyses of studies that evaluate health care interventions: explanation and elaboration. J Clin Epidemiol.

[36] Frank SR (2000). Digital health care--the convergence of health care and the Internet. J Ambul Care Manage.

[37] Braun V, Clarke V (2006). Using thematic analysis in psychology. Qual Res Psychol.

[38] Aqil A, Lippeveld T, Hozumi D (2009). PRISM framework: a paradigm shift for designing, strengthening and evaluating routine health information systems. Health Policy Plan.

[39] Michie S, van Stralen MM, West R (2011). The behaviour change wheel: a new method for characterising and designing behaviour change interventions. Implement Sci.

[40] Swanepoel DW, Hall 3rd JW (2010). A systematic review of telehealth applications in audiology. Telemed J E Health.

[41] Kuijpers W, Groen WG, Aaronson NK, van Harten WH (2013). A systematic review of Web-based interventions for patient empowerment and physical activity in chronic diseases: relevance for cancer survivors. J Med Internet Res.

[42] Morrison D, Wyke S, Agur K, Cameron EJ, Docking RI, Mackenzie AM, McConnachie A, Raghuvir V, Thomson NC, Mair FS (2014). Digital asthma self-management interventions: a systematic review. J Med Internet Res.

[43] Jalil S, Myers T, Atkinson I (2015). A meta-synthesis of behavioral outcomes from telemedicine clinical trials for type 2 diabetes and the Clinical User-Experience Evaluation (CUE). J Med Syst.

[44] Werder M (2015). Health information technology: a key ingredient of the patient experience. Patient Exp J.

[45] Jones L, Grech C (2016). The patient experience of remote telemonitoring for heart failure in the rural setting: a literature review. Contemp Nurse.

[46] Stokke R (2016). The personal emergency response system as a technology innovation in primary health care services: an integrative review. J Med Internet Res.

[47] Greenhalgh T, A'Court C, Shaw S (2017). Understanding heart failure; explaining telehealth - a hermeneutic systematic review. BMC Cardiovasc Disord.

[48] Sakaguchi-Tang DK, Bosold AL, Choi YK, Turner AM (2017). Patient portal use and experience among older adults: systematic review. JMIR Med Inform.

[49] Slater H, Campbell JM, Stinson JN, Burley MM, Briggs AM (2017). End user and implementer experiences of mHealth technologies for noncommunicable chronic disease management in young adults: systematic review. J Med Internet Res.

[50] Wildenbos GA, Peute L, Jaspers M (2018). Aging barriers influencing mobile health usability for older adults: a literature based framework (MOLD-US). Int J Med Inform.

[51] Ames HM, Glenton C, Lewin S, Tamrat T, Akama E, Leon N (2019). Clients' perceptions and experiences of targeted digital communication accessible via mobile devices for reproductive, maternal, newborn, child, and adolescent health: a qualitative evidence synthesis. Cochrane Database Syst Rev.

[52] Cheung KL, Durusu D, Sui X, de Vries H (2019). How recommender systems could support and enhance computer-tailored digital health programs: a scoping review. Digit Health.

[53] De La Cruz Monroy MF, Mosahebi A (2019). The use of smartphone applications (apps) for enhancing communication with surgical patients: a systematic review of the literature. Surg Innov.

[54] Lim S, Tan A, Madden S, Hill B (2019). Health professionals' and postpartum women's perspectives on digital health interventions for lifestyle management in the postpartum period: a systematic review of qualitative studies. Front Endocrinol (Lausanne).

[55] Palacholla RS, Fischer N, Coleman A, Agboola S, Kirley K, Felsted J, Katz C, Lloyd S, Jethwani K (2019). Provider- and patient-related barriers to and facilitators of digital health technology adoption for hypertension management: scoping review. JMIR Cardio.

[56] Brigden A, Anderson E, Linney C, Morris R, Parslow R, Serafimova T, Smith L, Briggs E, Loades M, Crawley E (2020). Digital behavior change interventions for younger children with chronic health conditions: systematic review. J Med Internet Res.

[57] Eze ND, Mateus C, Cravo Oliveira Hashiguchi T (2020). Telemedicine in the OECD: an umbrella review of clinical and cost-effectiveness, patient experience and implementation. PLoS One.

[58] Ingemann C, Hansen NF, Hansen NL, Jensen K, Larsen CV, Chatwood S (2020). Patient experience studies in the circumpolar region: a scoping review. BMJ Open.

[59] Wei Y, Zheng P, Deng H, Wang X, Li X, Fu H (2020). Design features for improving mobile health intervention user engagement: systematic review and thematic analysis. J Med Internet Res.

[60] Memon M, Wagner SR, Pedersen CF, Beevi FH, Hansen FO (2014). Ambient assisted living healthcare frameworks, platforms, standards, and quality attributes. Sensors (Basel).

[61] Firth J, Torous J (2015). Smartphone apps for schizophrenia: a systematic review. JMIR Mhealth Uhealth.

[62] Liddy C, Drosinis P, Keely E (2016). Electronic consultation systems: worldwide prevalence and their impact on patient care-a systematic review. Fam Pract.

[63] Morton K, Dennison L, May C, Murray E, Little P, McManus RJ, Yardley L (2017). Using digital interventions for self-management of chronic physical health conditions: a meta-ethnography review of published studies. Patient Educ Couns.

[64] Rincon E, Monteiro-Guerra F, Rivera-Romero O, Dorronzoro-Zubiete E, Sanchez-Bocanegra CL, Gabarron E (2017). Mobile phone apps for quality of life and well-being assessment in breast and prostate cancer patients: systematic review. JMIR Mhealth Uhealth.

[65] Lattie EG, Adkins EC, Winquist N, Stiles-Shields C, Wafford QE, Graham AK (2019). Digital mental health interventions for depression, anxiety, and enhancement of psychological well-being among college students: systematic review. J Med Internet Res.

[66] Wesselman LM, Hooghiemstra AM, Schoonmade LJ, de Wit MC, van der Flier WM, Sikkes SA (2019). Web-based multidomain lifestyle programs for brain health: comprehensive overview and meta-analysis. JMIR Ment Health.

[67] Choi W, Wang S, Lee Y, Oh H, Zheng Z (2020). A systematic review of mobile health technologies to support self-management of concurrent diabetes and hypertension. J Am Med Inform Assoc.

[68] O'Keefe M, White K, Jennings JC (2021). Asynchronous telepsychiatry: a systematic review. J Telemed Telecare.

[69] Cox A, Lucas G, Marcu A, Piano M, Grosvenor W, Mold F, Maguire R, Ream E (2017). Cancer survivors' experience with telehealth: a systematic review and thematic synthesis. J Med Internet Res.

[70] Barken TL, Söderhamn U, Thygesen E (2019). A sense of belonging: a meta-ethnography of the experience of patients with chronic obstructive pulmonary disease receiving care through telemedicine. J Adv Nurs.

[71] Walker RC, Tong A, Howard K, Palmer SC (2019). Patient expectations and experiences of remote monitoring for chronic diseases: systematic review and thematic synthesis of qualitative studies. Int J Med Inform.

[72] Leonardsen AL, Hardeland C, Helgesen AK, Grøndahl VA (2020). Patient experiences with technology enabled care across healthcare settings- a systematic review. BMC Health Serv Res.

[73] Steindal SA, Nes AA, Godskesen TE, Dihle A, Lind S, Winger A, Klarare A (2020). Patients' experiences of telehealth in palliative home care: scoping review. J Med Internet Res.

[74] Barello S, Triberti S, Graffigna G, Libreri C, Serino S, Hibbard J, Riva G (2016). eHealth for patient engagement: a systematic review. Front Psychol.

[75] Feather JS, Howson M, Ritchie L, Carter PD, Parry DT, Koziol-McLain J (2016). Evaluation methods for assessing users' psychological experiences of Web-based psychosocial interventions: a systematic review. J Med Internet Res.

[76] Baumel A, Birnbaum ML, Sucala M (2017). A systematic review and taxonomy of published quality criteria related to the evaluation of user-facing eHealth programs. J Med Syst.

[77] Rising KL, Ward MM, Goldwater JC, Bhagianadh D, Hollander JE (2018). Framework to advance oncology-related telehealth. JCO Clin Cancer Inform.

[78] Bashi N, Fatehi F, Mosadeghi-Nik M, Askari MS, Karunanithi M (2020). Digital health interventions for chronic diseases: a scoping review of evaluation frameworks. BMJ Health Care Inform.

[79] Lemon C, Huckvale K, Carswell K, Torous J (2020). A narrative review of methods for applying user experience in the design and assessment of mental health smartphone interventions. Int J Technol Assess Health Care.

[80] Søgaard Neilsen A, Wilson RL (2019). Combining e-mental health intervention development with human computer interaction (HCI) design to enhance technology-facilitated recovery for people with depression and/or anxiety conditions: an integrative literature review. Int J Ment Health Nurs.

[81] Molina-Recio G, Molina-Luque R, Jiménez-García AM, Ventura-Puertos PE, Hernández-Reyes A, Romero-Saldaña M (2020). Proposal for the user-centered design approach for health apps based on successful experiences: integrative review. JMIR Mhealth Uhealth.

[82] Fouquet SD, Miranda AT (2020). Asking the right questions-human factors considerations for telemedicine design. Curr Allergy Asthma Rep.

[83] Chaudhry H, Nadeem S, Mundi R (2021). How satisfied are patients and surgeons with telemedicine in orthopaedic care during the COVID-19 pandemic? A systematic review and meta-analysis. Clin Orthop Relat Res.

[84] Blessing LT, Chakrabarti A (2009). DRM, a Design Research Methodology.

[85] (2015). Design methods for developing services. Design Council.

[86] Ball J (2019). The Double Diamond Process Model. Design Council.

[87] Slater H, Briggs A, Stinson J, Campbell JM (2017). End user and implementer experiences of mHealth technologies for noncommunicable chronic disease management in young adults: a qualitative systematic review protocol. JBI Database System Rev Implement Rep.

[88] Dekkers T (2020). Data-driven Patient Profiles: definition, validation, and implementation for tailored orthopaedic healthcare services. Delft University of Technology.

[89] PatientsLikeMe.

[90] Wicks P, Mack Thorley E, Simacek K, Curran C, Emmas C (2018). Scaling PatientsLikeMe via a "generalized platform" for members with chronic illness: Web-based survey study of benefits arising. J Med Internet Res.

[91] Vagal A, Wahab S, Lecky S, Washburn E, Schwartz R, Vogel C, Mahoney M (2020). Optimizing patient experience using human-centered design. J Am Coll Radiol.

[92] Yao R, Zhang W, Evans R, Cao G, Rui T, Shen L (2022). Inequities in health care services caused by the adoption of digital health technologies: scoping review. J Med Internet Res.

[93] Zanaboni P, Ngangue P, Mbemba GI, Schopf TR, Bergmo TS, Gagnon MP (2018). Methods to evaluate the effects of Internet-based digital health interventions for citizens: systematic review of reviews. J Med Internet Res.

[94] Gorst SL, Armitage C, Hawley M, Coates E (2013). Exploring patient beliefs and perceptions about sustained use of telehealth. Int J Integr Care.

[95] Sanders C, Rogers A, Bowen R, Bower P, Hirani S, Cartwright M, Fitzpatrick R, Knapp M, Barlow J, Hendy J, Chrysanthaki T, Bardsley M, Newman SP (2012). Exploring barriers to participation and adoption of telehealth and telecare within the Whole System Demonstrator trial: a qualitative study. BMC Health Serv Res.

