# Supplementary material for: Digital Patient Experience: Umbrella Systematic Review
Source: J Med Internet Res. 2022 Aug 4;24(8):e37952. doi: 10.2196/37952 (PMC9389377; doi:10.2196/37952)
Supplement: Multimedia Appendix 1 [file jmir_v24i8e37952_app1.docx]

Appendix 1. Study Characteristics and Digital Health Interventions Characteristics of Included Reviews.

| **Reference** | **Research Aims or Questions** | **Review Types, Analysis Methods and Included Studies** | **Populations & Health Issues** | **Digital Health Interventions** |
| --- | --- | --- | --- | --- |
|  |  |  |  |  |
| **1.Swanepoel and Hall III 2010** [40] | To review audiology-related telehealth services and patient & clinician perceptions regarding its use. | Systematic review;  26 | Patients and clinicians related to audiology. | Telehealth applications (in a combination of synchronous, asynchronous, and hybrid models) for screening, diagnosis, and intervention. |
| **2.Kuijpers, Groen et al. 2013** [41] | To systematically review the effects of interactive Web-based interventions aimed at increasing patient empowerment and physical activity for various chronic conditions, and to explore their possible relevance for cancer survivors. | Systematic review;  19. | Patients with diabetes, chronic obstructive pulmonary disease (COPD), (congestive) heart failure, cardiovascular disease, and cancer. | Web-based health intervention for education, self-monitoring, feedback or tailored information, self-management training, personal exercise program, and communication. |
| **3.Memon, Wagner et al. 2014** [60] | To provide a comprehensive review of the Ambient Assisted Living (AAL) field with a focus on healthcare frameworks, platforms, standards, and quality attributes. | Literature review (& Email-Based Survey).  A state-of-the-art survey analysis;  113 | Elderly populations. | Ambient assisted living (AAL) systems |
| **4.Morrison, Wyke et al. 2014** [42] | To summarize the effectiveness and implementation of digital self-management support for adults and children with asthma and to examine what features help or hinder its use. | Systematic Review;  10 | Adults and children with asthma | Digital interventions (computer game or program, CD-ROM, Internet enabled device or CD-ROM, Web-based) for self-management |
| **5.Brunton, Bower et al. 2015** [28] | To systematically review user perspectives and experiences of telehealth in COPD (Chronic Obstructive Pulmonary  Disease) management, in order to identify factors that may impact uptake. | Systematic review.  Meta-synthesis;  10. | People with chronic obstructive pulmonary disease (COPD) | Telehealth for monitoring patients’ physiological signs, Self-management. |
| **6.Firth and Torous 2015** [61] | To review the published literature of smartphone apps applied for the care of patients with schizophrenia and other psychotic disorders. | Systematic review;  7. | People with psychotic disorders and schizophrenia. | Smartphone apps for self-assessments, symptom monitoring, self-management, physical activity promotes. |
| **7.Jalil, Myers et al. 2015** [43] | To review the effectiveness and the provided improvements of telemedicine services for the treatment of type 2 diabetes. | Systematic review;  Meta-synthesis;  9. | People with type 2 diabetes. | Telemedicine (Telephone-based, computer-based, and hand-held) for behaviour changes and chronic illness management. |
| **8.Werder 2015** [44] | To understand the linkages of health information technology (HIT) and patient experience. | Literature review;  11. | N/A | Health information technology (computers, mobile devices, software, and various communication devices). |
| **9.Barello, Triberti et al. 2016** [74] | To review findings from the literature about the use of eHealth in engaging patients in their own care process. | Systematic review (PRISMA ^a^);  11. | N/A | eHealth intervention for patient engagement. |
| **10.Feather, Howson et al. 2016** [75] | To investigate what methods, if any, have been used to evaluate the in situ psychological experience of users of Web-based self-help psychosocial interventions. | Systematic review (AMSTAR ^b^);  21. | People with psychological issues | Web-based psychosocial interventions for Self-help. |
| **11.Jones and Grech 2016** [45] | To examine the available evidence and inform the development of a telemonitoring service. | Literature review;  Thematic analysis;  11. | Community dwelling people over the age 18 with heart failure living in the rural and remote Queensland. | Remote telemonitoring for disease management. |
| **12.Liddy, Drosinis et al. 2016** [62] | To understand the effectiveness, population impact and costs associated with implementation of eConsult services. | Systematic review (PRISMA ^a^);  Thematic analysis;  36. | N/A | Electronic consultations system (an asynchronous, directed communication over a secure electronic medium) for communication between primary care providers and specialist physicians. |
| **13.Stokke 2016** [46] | To explore how different actors experience the technology in use of PERS (The Personal Emergency Response System) and how it affects the complex interactions between multiple actors in caring practices. | Integrative review;  Descriptive, integrative, thematic analysis;  33. | Older people | The personal emergency response system in home care. |
| **14.Baumel, Birnbaum et al. 2017** [76] | To identify and classify key criteria concepts related to the evaluation of user-facing eHealth programs. | Systematic review (PRISMA ^a^); Taxonomy;  84. | N/A | User-facing eHealth. |
| **15.Cox, Lucas et al. 2017** [69] | To characterize the patient experience of telehealth interventions for adult cancer survivors. | Systematic review (ENTREQ ^c^);  Thematic synthesis;  22. | Adult cancer survivors aged 18 years and over. | Telehealth intervention (telephone, Internet, or hand-held or mobile technology) for remote communication or remote monitoring with an HCP. |
| **16.Greenhalgh and Shaw 2017** [47] | To inform policy by making sense of a complex literature on heart failure and its remote management. | Systematic review;  Hermeneutic synthesis;  105. | Patients, staff and organisations related to heart failure. | Telehealth (telephone, tele-monitoring devices, interactive portals, video) for remote management. |
| **17.Morton, Dennison et al. 2017** [63] | To understand the experiences of patients and healthcare professionals (HCPs) using self-management digital interventions (DIs) for chronic physical health conditions. | Systematic review. (ENTREQ ^c^ & PRISMA ^a^); Meta-ethnography synthesis;  30. | Patient and healthcare professionals related to chronic physical health conditions. | Digital interventions for self-management. |
| **18.Rincon, Monteiro-Guerra et al. 2017** [64] | To (1) identify evidence-based mobile phone health apps focused on QoL and well-being (anxiety and depression symptoms) in breast or prostate cancer patients, (2) recognize their clinical and technological characteristics, (3) categorize their clinical and technological strengths and weaknesses, and (4) determine patients’ user experience (satisfaction level and comments regarding the apps used) | Systematic review (PRISMA ^a^);  5. | Patients with breast and prostate cancer. | Mobile phone apps. |
| **19.Sakaguchi-Tang, Bosold et al. 2017** [48] | To assess the existing research landscape related to patient portal and electronic personal health records (ePHRs) use and experience among older adults and to understand the benefits and barriers to older adults’ use and adoption of patient portals and ePHRs. | Systematic review (PRISMA ^a^);  17. | Older adults aged 60 years or over. | Patient portals and electronic personal health records (ePHRs) |
| **20.Slater, Campbell et al. 2017** [49] | To identify, appraise, and synthesize available qualitative evidence on users’ experiences of mHealth technologies for chronic noncommunicable diseases (NCD) management in young people. | Systematic review (PRISMA ^a^ & ENTREQ ^c^);  12. | End users (young people in the age range of 15-24 years) and implementers (health policy makers, clinicians, and researchers) related to chronic noncommunicable diseases (NCDs). | mHealth technologies (mobile device or service) for self-management. |
| **21.Rising, Ward et al. 2018** [77] | To identify and categorize within the the National Quality Forum (NQF) domains currently existing measures of telehealth applicable to oncology to detect priority areas for future research and measure development. | Review of systematic review;  Meta-analyses;  12. | Oncology patients with cancer. | Telehealth applications. |
| **22.Wildenbos, Peute et al. 2018** [50] | To synthesize literature on aging barriers to digital (health) computer use, and explain, map and visualize these barriers in relation to the usability of mHealth by means of a framework. | Scoping review; Thematic analysis;  23. | Older adults aged 50 years or over. | Digital (mobile) health applications. |
| **23.Ames, Glenton et al. 2019** [51] | To explore clients' perceptions and experiences of targeted digital communication via mobile devices on topics related to reproductive, maternal, new-born, child, or adolescent health (RMNCAH). | Systematic review; Qualitative evidence synthesis;  35. | Clients related to reproductive, maternal, newborn, child, or adolescent health. | Targeted digital communication accessible via mobile devices in the areas of RMNCAH. |
| **24.Barken, Söderhamn et al. 2019** [70] | To synthesize the qualitative research in the literature addressing how patients with chronic obstructive pulmonary disease (COPD) experience care received by telemedicine. | Systematic review;  Meta-ethnography (The ENTREQ ^c^ and eMERGe guidelines ^d^);  66. | Patients with chronic obstructive pulmonary disease care (COPD). | Telemedicine technologies for information, communication, and monitoring. |
| **25.Cheung, Durusu et al. 2019** [52] | To explore: (1) how recommender systems provide health recommendations, (2) to what extent recommender systems incorporate theoretical models and (3) how the use of recommender systems may enhance the usage of computer-tailored interventions. | Scoping review (Arksey and O’Malley’s five steps of scoping review methodology);  26. | N/A | Computer-tailored digital health program (recommender systems). |
| **26.De La Cruz Monroy and Mosahebi 2019** [53] | To carry out a literature review of studies that evaluate patients’ experience on the use of perioperative mHealth apps. | Systematic review (PRISMA ^a^);  11. | Surgical patients of perioperative. | Perioperative mHealth apps for patient-to-provider communication. |
| **27.Lattie, Adkins et al. 2019** [65] | To identify the effectiveness, usability, acceptability, uptake, and adoption of digital mental health interventions focused on depression, anxiety, and enhancement of psychological well-being among college students. | Systematic review (PRISMA ^a^);  89. | College students’ mental health (depression and anxiety) & psychological well-being | Digital health (Web-based technology, Mobile phone) |
| **28.Lim, Tan et al. 2019** [54] | To explore postpartum women and health professionals’ perspectives of digital health interventions (DHIs) for lifestyle management in postpartum wome. | Systematic review (PRISMA ^a^);  Thematic synthesis;  9. | Health professionals and postpartum women in the postpartum period. | Digital health intervention for lifestyle management. |
| **29.Palacholla, Fischer et al. 2019** [55] | To provide a comprehensive summary of provider- and patient-related barriers to and facilitators of digital health technology (DHT) adoption for hypertension management. | Scoping review (Arksey and O’Malley’s five steps of scoping review methodology);  36. | Providers and patients of hypertension management. | Digital health technology for management. |
| **30.Søgaard Neilsen and Wilson 2019** [80] | To investigate how can human computer interaction facilitate a positive experience for people with depression and anxiety. | Integrative review (PRISMA ^a^);  30. | People with depression or anxiety conditions. | eHealth intervention (online therapeutic psychoeducational interventions). |
| **31.Walker, Tong et al. 2019** [71] | To describe the range of patients’ beliefs, attitudes, expectations, and experiences of remote monitoring for chronic conditions across different healthcare contexts and populations. | Systematic review (ENTREQ ^c^); Thematic synthesis;  16. | Adult patients (over 18 years) with any chronic diseases. | Remote monitoring. |
| **32.Wesselman, Hooghiemstra et al. 2019** [66] | to (1) provide a comprehensive overview of Web-based multidomain lifestyle programs aimed at optimizing brain health in healthy adult populations and (2) ​describe the programs and targeted lifestyle factors, availability, and evaluation of adherence, user experience and effectiveness. | Comprehensive Overview (PRISMA ^a^); Meta-Analysis;  44. | Healthy adult population (for brain health) | Web-based multidomain lifestyle programs (website, Web application or app) and self-administered with no need for a visit to or from a health care professional). |
| **33.Bashi, Fatehi et al. 2020** [78] | To explore the nature, extent and components of existing digital health (DH) frameworks for chronic diseases. | Scoping review (Arksey and O’Malley’s five steps of scoping review methodology);  11. | Patients with chronic diseases | Digital health intervention (digital, mobile and wireless technologies). |
| **34.Brigden, Anderson et al. 2020** [56] | To (1) identify effective digital interventions, (2) report the characteristics of promising interventions, and (3) describe the user’s experience of the digital intervention | Systematic Review (PRISMA ^a^);  17. | Younger children aged between 5 and 12 years with chronic health conditions. | Stand-alone or guided digital health intervention (internet, personal computers, social media, mobile phones, or smartphones) for behaviour change. |
| **35.Choi, Wang et al. 2020** [67] | To (1) characterize mHealth technologies used or described in the mHealth literature and (2) summarize their effects on self-management for people with diabetes and hypertension from the clinical and technical standpoints. | Systematic review (PRISMA ^a^);  11. | Patients with concurrent diabetes and hypertension. | Mobile health technologies (mHealth apps, Web interfaces, Bluetooth-enabled devices) for self-management, clinical decision and shared decision making. |
| **36.Eze, Mateus et al. 2020** [57] | To provide summary evidence of telemedicine's effectiveness, cost-effective, patient experiences and implementation. | Umbrella review (A prior written, unregistered protocol);  98. | People from countries within the Organisation for Economic Co-operation and Development (OECD). | Telemedicine (remote monitoring, real-time, and store-and-forward) for either synchronous or asynchronous communication between a patient and their health care workers (HCWs). |
| **37.Fouquet and Miranda 2020** [82] | To provide a narrative review of human factors considerations for telemedicine. | Narrative review;  26. | N/A | Telemedicine. |
| **38.Ingemann, Hansen et al. 2020** [58] | To describe the range and nature of peer-reviewed literature on patient experience studies conducted within the circumpolar region. | Scoping review (Arksey and O’Malley’s five steps of scoping review methodology & PRISMA ^a^);  96. | People living in the circumpolar region, which is home to many Indigenous populations. | Telehealth from a specific illness to health pro- motion activities. |
| **39.Lemon, Huckvale et al. 2020** [79] | To characterize and compare user experience (UX) evaluation approaches that have been applied in mental health smartphone interventions, and to identify implications for research and practice. | Narrative Review. | Mental health. | Smartphone intervention. |
| **40.Leonardsen, Hardeland et al. 2020** [72] | To summarize empirical studies exploring patient experiences with Technology Enabled Care (TEC). | Systematic review (PRISMA ^a^);  21. | Patients (aged 18 years over) related to somatic diseases, treatment and care across healthcare settings. | Technology enabled care in all healthcare settings. |
| **41.Molina-Recio, Molina-Luque et al. 2020** [81] | To propose a methodology based on the review of previous successful user experiences in setting up health apps by using qualitative techniques that includes the participation of information technology and health professionals and the patients themselves. | Integrative review;  68. | The participation of information technology, health professionals and patients. | Health apps. |
| **42.Steindal, Nes et al. 2020** [73] | To map and assess published studies on the use of telehealth for patients in palliative home care. | Scoping review (Arksey and O’Malley’s five steps of scoping review methodology & PRISMA-ScR ^e^);  22. | Patients in a palliative care trajectory regardless of diagnosis, aged 18 years or older, and living at home. | Telehealth (at home with follow-up from health care professionals). |
| **43.Wei, Zheng et al. 2020** [59] | To investigate which design feature improves user engagement with mHealth interventions. | Systematic review (PRISMA ^a^); Thematic analysis;  35. | General populations. | Mobile health (Mobile phone app, Website platform, text messages). |
| **44.Chaudhry, Nadeem et al. 2021** [83] | To investigate (1) what are the levels of patient and surgeon satisfaction with the use of telemedicine as a tool for orthopaedic care delivery? (2) Are there differences in patient-reported outcomes between telemedicine visits and in-person visits? (3) What is the difference in time commitment between telemedicine and in-person visits? | Systematic review (PRISMA ^a^); Meta-analysis;  12. | Patients and surgeons related to orthopaedic care during COVID-19 pandemic. | Telemedicine (Phone, Video, Internet-based) for consultation or follow-up visits. |
| **45.O’Keefe, White et al. 2021** [68] | To assess existing asynchronous telepsychiatry (ATP) research according to the telehealth domains established by The National Quality Forum (NQF), evaluates the prevalence and quality of ATP, and identifies the areas in which more research must be conducted. | Systematic review (PRISMA ^a^);  11. | Psychiatry. | Asynchronous telehealth related to ATP intervention (transmission of psychiatric clinical information through web technologies from a patient or doctor to a remote specialist). |

^a^PRISMA: the Preferred Reporting Items for Systematic Reviews and Meta-Analyses (PRISMA) guidelines.

^b^ AMSTAR: Assessment of Multiple Systematic Reviews.

^c^ ENTREQ: the Enhancing Transparency in Reporting the Synthesis of Qualitative Research (ENTREQ) framework.

^d^ eMERGe: The eMERGe meta-ethnography reporting guidance.

^e^ PRISMA-ScR: the Preferred Reporting Items for Systematic Reviews and Meta-Analyses extension for Scoping Reviews (PRISMA-ScR) checklist.
