# Supplementary material for: Digital Patient Experience: Umbrella Systematic Review
Source: J Med Internet Res. 2022 Aug 4;24(8):e37952. doi: 10.2196/37952 (PMC9389377; doi:10.2196/37952)
Supplement: Multimedia Appendix 2 [file jmir_v24i8e37952_app2.docx]

#### Multimedia Appendix 2. Influencing factors on digital patient experience (double-edged factors imply diverse impact, positive factors imply positive impact, and negative factors imply negative impact).

| Themes | | | Studies, n (%) | Positive factors | Negative factors | Double-edged factors | References |
| --- | --- | --- | --- | --- | --- | --- | --- |
| **Behavioral determinants** **(3 categories, 6 themes, 11 positive factors, 21 negative factors, and 5 double-edged factors)** | | | | | | | |
|  | **Patient capability category** | | | | | | |
|  |  | Knowledge and skills | 16 (36) | - Familiarity with the technology - Previous positive experience with digital health | - Low literacy (language, technology; or health) - Previous negative experiences with digital health (eg, failure to achieve goals and disappointment with the DHIs^a^) | N/A^b^ | [28,43,45-48,51,53,56,57,63,64,69,71,73,80] |
|  |  | Confidence levels | 6 (13) | N/A | - Lack of confidence in skills - Perceived inability to use technologies or services - Misunderstanding digital health tasks | N/A | [43,45,47,48,71,81] |
|  | **Patient opportunity category** | | | | | | |
|  |  | Identity | 22 (49) | - Younger age (eg, easier access to the internet) - DHIs fit into patient’s daily routine | - Older age (eg, with age-related barriers) - Low socioeconomic status (eg, lack of access to digital health) - Business (eg, travel required, household responsibilities) | - Gender differences | [28,40,42,43,46,48-51,53-57,61,63,64,66,69,71-73] |
|  |  | Health status | 20 (44) | - The earlier stages of illness and partial or full remission | - Advanced chronic disease and complex comorbidities - The acute stages of illness - Out of control health condition - Cognitive barriers | - A stable (ie, under control) health condition | [28,43,45-50,53,55-57,61,63,69,70,73,76,81,82] |
|  | **Patient motivation category** | | | | | | |
|  |  | Perception (perceived advantages and disadvantages) | 21 (47) | - Perceived sense of security, independence, empowerment, convenience and access to care, and less sense of vulnerability - Prepared for emergencies or hospital visits - Intact social networks - A suitable goal setting | - Perceived no benefits - Perceived threats to security, privacy, independence, or an individual’s sense of identity - Worried digital health would replace traditional appropriate (face-to-face) health care services - Impede social life or interfere with patient-to-provider relationships - Cause additional burden (being bombarded with too many messages) | N/A | [28,41,43,45-51,54,55,57,60,61,63,66,69,71,76,81] |
|  |  | Mindset | 15 (33) | - Prefer digital solutions - Strong desire to keep healthy or gain knowledge | - Computer anxiety - Overreliance - Loss of interest - Lack of motivation | - Patient preferences, expectations, desires, priorities, understanding, or beliefs - Reliance - Trust in technology | [28,40,42,43,45,47,48,51,54,57,63,69,71,73,81] |
| **Technical determinants (3 categories, 13 themes, 59 positive factors, 35 negative factors, and 13 double-edged factors)** | | | | | | | |
|  | **Intervention technology category** | | | | | | |
|  |  | Technical usability | 31 (69) | - Ease of use and understanding - Ready-to-use applications and devices - Automatic and seamless system updating - Adaptive interface - Avoiding error prompts | - Difficulty to use - Equipment or battery failure - High system complexity (eg, complex software downloads and account or password settings) - Data transmission and input difficulties - Unstable internet connection or slow loading of website - Low accessibility - Low error tolerance - Poor picture and sound quality - Low visibility on small screens | N/A | [28,41,43-49,51,53-60,64,66,67,69,70,72,73,76,78-82] |
|  |  | Technical features | 23 (51) | - Detect an improvement from digital health data or share data with HCPs^c^ - Medication or appointment reminders or altering - Symptoms tracking dairies or tools - Timely feedback or motivational feedback notifications - Ability to print or email information - Ability to take voice commands - Nutrition calculator - Clinical measurements - A security password for record access - Agenda setting - Recommender systems - Summary reports for supporting shared decision-making - Input or review information at any point | - Access to changeless or worse physiological data over time | - Access to data | [28,41,42,44,48,49,51,52,54-57,59,63,64,66,67,69-71,73,80,81] |
|  |  | Delivery media or devices | 14 (31) | - Mobile technology - Video- or audio-based technology (for users with sensory impairments) - Assisted equipment (eg, provide headphones for people with hearing difficulties and larger monitors with improved lighting for people with visual impairments) | - Web-based technology | - Types of devices (eg, mobile phones or computers) - Device ownership (eg, personal devices or devices without personal identifiers) - Types of channels (eg, SMS text message or videos) | [28,49,51,53-55,57,61,64,70,72,73,81,82] |
|  | **Intervention functionality category** | | | | | | |
|  |  | Intervention goals | 30 (67) | - Individualized or timely feedback - Remotely consultation with HCPs - Provide sufficient health information | - Be forced to share data with HCPs, which is undesired by patient - Under long-term video-based monitoring | - Remote data monitoring - Self-management support - Health information provision and patient education - Shared decision-making | [28,41-46,48,49,51,53-57,60,61,63,64,66,67,69-73,76,78,80,81] |
|  |  | Social support | 23 (51) | - Interact with a real human being - Regular and continuous patient-to-physician interaction - Connect with peers - Exchange health information and advice with family and friends | - Replace interpersonal connections with HCPs - Lack of physical human contact with HCPs - Unable to contact HCPs directly or obtain timely feedback | - Remote connection | [28,41,43,44,46,48,51-60,66,69-71,73,76,78] |
|  |  | Performed quality | 19 (42) | - Reliability and credibility (eg, owner’s credibility, maintenance, third party verification, research support, involvement of clinical experts in the design process, and empirical evidence for successful implementation) - Regulation compliance - Flexibility | - Less accuracy of clinical assessments - Lack of availability and accessibility - Lack of safety and privacy (eg, incorrect intervention dosage and the absence of privacy notifications) - Without well-defined or safely standardized clinical indicators | N/A | [28,41,45,47-49,51,53-55,57,59-61,64,69,76,78,81] |
|  |  | Intervention structure | 12 (27) | - A structured format or regular weekly contact with HCPs - Longer duration - Flexible interventions | - Structured interventions not tailored to patients’ individual symptoms and preferences - Structured interventions that constantly remind patients of their symptoms | - The intensity, frequency or duration of interventions - Prefixed interventions | [28,41,51,53-55,57,64,66,69,70,81] |
|  |  | Theoretical background | 11 (24) | - Presence of multiple underlying theories (BCTs^d^, EBIs^e^, and persuasive technology) | N/A | N/A | [42,43,53,54,56,57,59,66,76,78,80] |
|  | **Intervention interaction design category** | | | | | | |
|  |  | Personalized design | 23 (51) | - Individualized feedback, tailored features, or customization - Be able to choose the topic, content, and language of received messages - Be able to select the timing and frequency of the delivered interventions | N/A | N/A | [41-43,45,49,51-57,59,60,63,64,69,70,72,76,78, 80,81] |
|  |  | Design procedures | 22 (49) | - User-centered design or human-centered design - Interorganizational collaboration - Co-design or participatory development methodology - Inclusive design - Involvement of multistakeholder and multidisciplinary teams in the early design stages | N/A | N/A | [44,45,47-50,55-57,60,64-66,69,71,72,75,76,78,80-82] |
|  |  | Navigation design | 18 (40) | - Instruction manuals and extra user training - Technical support or assistance - Interactive elements | - Lack of clear navigation or instruction design | N/A | [43,45,48,49,53-55,57,59,60,63,66,71,72,76,80-82] |
|  |  | Visual design | 15 (33) | - Visualized health data - Tailored, attention-grabbing, simple, and consistent layout design (eg, appealing graphic presentation, pleasing and coherent color scheme, high text quantity, suitable font and interface size, and striking button appearance and location) - Unobtrusive wearable devices | - Unappealing user interfaces - Poorly crafted interface - Low visibility of the content - Bulkiness - Nonportability - Small screen or font size | NA | [43,54,57,59,60,63,64,66,67,72,73,76,80-82] |
|  |  | Information design | 12 (27) | - A reliable, trusted, credible information source - An unmarked sender - Multimedia messages - Detailed and comprehensive information - Diverse and updated information - A short, concise, personalized, clear, and direct message - Formal or clinical language for some functions (description of pathologies) - Informal language for others (evaluation of conduct) - A motivational, friendly, encouraging, polite, respectful, congratulatory, personalized, upbeat, positive, humorous, and relatable tone - Layered medication information and warnings from basic to advanced | - Overload of information - Technical language | - Information source - Information language | [45,48,51-54,59,66,73,76,81,82] |
| **Organizational determinants (3 categories, 5 themes, 13 positive factors, and 23 negative factors)** | | | | | | | |
|  | **Organizational environment category** | | | | | | |
|  |  | Cost and time | 21 (47) | - Less travel costs and waiting time - Complete tasks at patients’ own pace - Less time consuming - Faster response - Real-time feedback or timely support | - High start-up costs, ongoing costs, and costs related to loss of revenue - The cost of damage to equipment - Unrealistic financial reimbursement and higher costs relevant to internet or equipment - Time consuming for daily monitoring or recharging devices - Energy to complete “one more task” - Disruption to the daily routine - Lack of timely feedback - Long waiting times for digital health calls | N/A | [42,45-49,51,53-58,60,64,69-72,81,83] |
|  |  | Health care providers | 18 (40) | - Many more HCPs - Clinical positive opinion and approval - Polite attitudes - Good communication skills | - Clinician resistance (eg, less positive views on digital health) - Undermined clinical capacity and professional identity - Increased clinical workload - Impeded communication with patients - Overtreatment | N/A | [28,44-49,51,55-59,63,69,72,73,78,81] |
|  |  | Health information systems | 13 (29) | - The compatibility, interoperability, integration, sustainability, and completeness of systems - The clarity and transparency on accountability, workflow, and data processing - Clear information on required stakeholder responsibility | - Lack of compatibility and interoperability of the system with different mobile operating systems and terminals - Poor integration and working relationship between the service team - Lack of adequate installation - Connectivity issues between medical devices and mobile terminals - Limitation on scalability | N/A | [28,44,45,47,49,57,60,63,67,69,80-82] |
|  | **Physical environment category** | | | | | | |
|  |  | Place | 9 (20) | - Stay in a familiar and relaxing environment; not restricted to the hospital setting | - Environmental distractions (eg, background noise and lighting) | N/A | [48,49,51,57,58,69,72,73,82] |
|  | **Social environment category** | | | | | | |
|  |  | Culture | 4 (9) | N/A | - The absence of or inadequate supporting policies and legislation - Lack of a plausible business case - Unrealistic financial reimbursement - Lack of well-established sociotechnical infrastructure | N/A | [47,49,57,78] |
