# Supplementary material for: Digital Patient Experience: Umbrella Systematic Review
Source: J Med Internet Res. 2022 Aug 4;24(8):e37952. doi: 10.2196/37952 (PMC9389377; doi:10.2196/37952)
Supplement: Multimedia Appendix 3 [file jmir_v24i8e37952_app3.docx]

#### Appendix 3. Themes of influencing factors of the digital patient experience.

#### Patient Capability

The large heterogeneity between user populations leading to more complex UX and socio-technical requirements among DHI [40]. Some health professionals also questioned patients’ ability to perform tasks in telehealth [41]. Patient capability, including patients’ knowledge and skills on operating digital health technologies and confidence levels for completing digital health tasks. Knowledge and skills are more about patients’ previous experiences, including language literacy, technology literacy or health literacy, as well as their familiarity with digital health techniques. Familiarity with the technology can impact patient acceptance of digital health [28, 42, 43, 60, 61]. End users who have a positive previous experience on digital solutions normally were more compliant to and satisfied with DHI [44]. However, for those who did not experience benefits from using DHI, for instance, disappointment with the intervention [43] or failed to achieve goals [75], were less engaged in managing their condition [41]. The level of patients’ personal literacy limited their ability to access digital communication [45, 46, 60, 62, 76]. Inability to safely operate a DHI equipment was a barrier to patient acceptance and required patients to develop skills to use the equipment [28, 44, 47, 63, 69]. Confidence levels refers to patients’ perceived ability of operating DHI. Lack of confidence in own ability or perceived inability to use the technology or service [28, 46, 47, 64, 69, 74], which might result by misunderstanding the tasks in using the DHIs [69], leads to a negative digital PEx.

#### Patient Opportunity

Patient opportunity refers to their identity and health status. Identity contains patients’ demographic characteristics and daily routines. The level of acceptability varied by population demographics, such as age, gender, and social-economic status [48, 76]. The elderlies were less likely to be positive about DHIs [49, 74] and often experienced age-related barriers [77], including limited physical flexibility, reduced cognition, low technical literacy and sensory impairments, resulting in using difficulties of computer interaction (eg, accurately clicking buttons, making operation gestures or distinguishing icons on screens) [43, 46, 77]. On the contrast, younger patients, in their 20s and 30s, were easier to develop a trusting relationship with their health care providers [45, 50], access to and familiarity with the Internet. Gendered differences also resulted in different Internet access and equipment usage [44, 46, 60]. Lack of access to mobile devices were reported as barriers to using the DHI [60]. Patients valued DHI that is easy to integrate into their daily routines [51, 70], and it was perceived as convenient to use [45], leaded to patient satisfaction [50] and sustained platform use [52]. Travel required, household responsibilities or too busy caused dropout [48, 74]. Health status refers to patients physical, psychological and cognitive conditions [44, 46, 47, 69, 74]. Patients’ uptake of DHIs was impacted by the complexity, severity and stability of their illness [51, 53, 64-65, 69, 75]. Advanced chronic disease and complex comorbidities resulted in complex monitoring requirements, which increased the patients’ anxiety of using DHI [69]. Patients in the acute stages of illness may have more difficulty with adhering DHIs than those in the earlier stages of illness, partial or full remission [53]. Normally, patients in a stable health condition perceived less benefits from data monitoring [75] and considered DHIs restricted their daily life since it reminded them of their illness [65]. Patients with an increased symptom burden caused by out of control often found self-monitoring DHI to be a frustrating process [75]. Patient felt increased anxiety if they checked data too often [51, 70]. Patients with cognitive barriers (eg, poor memories) might result in usage difficulties (eg, unable to remember usernames and passwords) [43, 45, 46, 63, 70, 77].

#### Patient Motivation

Patient motivation of using DHI often resulted from their perceived advantages, perceived disadvantages, and initial mindset. Patients perceived advantages which stimulate them to use DHI [28, 51, 52, 75, 77]. Some patients reported DHIs bring them the sense of security [28, 41, 44, 60, 75], independence [41, 69, 74], empowerment [41, 51], convenience and access to care [60], preparation for emergencies or hospital visits [46], intact social networks [43] and less sense of vulnerability [45]. However, lack of motivation [47, 48, 65, 74, 76, 77], loss of interest [55], perceived non-benefits [47, 77], resulted in low patient adherence. A suitable goal setting, such as focusing on interventions’ duration rather than intensity might help patients keep motivated [75]. Perceived disadvantages often result in negative digital PEx or stop patients’ motivation of using DHI. Some patients perceived DHIs threat their security or privacy [40, 44, 46, 53, 55, 64, 70], replace traditional appropriate (face-to-face) health care [28, 44, 46-47, 60, 69], undermine independency or individual’s sense of identity [40, 41, 60, 69], impede social life or interfere with patient-provider relationship [28, 44, 70], and cause additional burden (eg, being bombarded with too much messages) [28, 41, 60]. Some patients even fear of DHI not working when they needed [44] and concern about technical operation complexity [48]. Patients’ mindset refers to patient preferences [44, 45, 47, 49, 60, 76, 80], expectations [41, 45, 60, 69, 75], reliance [41, 45, 60, 75], trust on technology [28], desires and priorities [48], computer anxiety [46], as well as understandings and beliefs [69] of DHIs. Patients’ mindsets varied and may result in diverse impacts. For instance, patients’ desire to keep healthy or gain knowledge may motivate them to use DHI [48]. However, their over-reliance on DHI may lead to a delayed help seeking and a negative digital PEx [41].

#### Intervention Technology

DHI is not a new type of health care, but rather a new technology for delivering already existing types of health care [71]. Intervention technology was one of the complex factors that impact digital PEx since it normally required the end-users who have no technical expertise, with disabilities or specific health deficiencies in handling different devices, applications, network equipment, gateways and other infrastructural components [40]. It refers to technical usability, technical features, and delivery media. Technical usability was defined as how easy the provided digital health technologies are to use based on Nielsen’s definition of usability [66]. Ease of use and understand [41, 43, 46, 48, 50-52, 54, 61, 64, 69, 70, 74, 78] can help to reduce patients' concerns. Ready-to-use applications and devices with automatic and seamless system updating, adaptive interface and avoiding error prompts can improve easy to use of DHI [40]. However, if the complexity of the technology higher than patients’ skill or confidence levels can inhibit their competence and willingness to use DHI [46, 74]. Difficulty to use [41, 47, 50, 61, 74, 76] normally resulted in patients’ feelings of confusion, frustration and failure. Equipment or battery failure [41, 44, 55, 60, 64, 65, 69], high system complexity [40, 41, 46, 69, 74], data transmission and input difficulties [46, 50, 56, 69, 76], instable Internet connection [45, 55, 64, 69, 70], complex software downloads, account or password settings [52, 70, 67], low accessibility [46, 60, 69], low error tolerance [40, 74], slow loading of website [54, 55], poor picture and sound quality [69], low visibility on the small screens [56] leads to difficulty of use.

Technical features refer to the unique interactive elements [57, 80] of DHI compared to the face-to-face healthcare systems, including reminders about medication, appointments and communication [42, 43, 46, 51, 52, 56, 60, 78], dairies or tools for tracking symptoms [42, 48, 51, 60, 61, 75], timely feedback or motivational feedback notifications [42, 56, 61, 62, 70], ability to print or email information and to take voice commands (data input) [46, 48, 51, 52, 56], medication altering [46, 56], nutrition calculator [51, 60], clinical measurements [56, 65], a security password for record access [46, 78], agenda setting [46, 51], recommender systems [82], summary reports for supporting shared decision-making [56] normally help to improve PEx. Being able to access to DHI data or sharing data with HCPs [28, 41, 65, 70], write or input communication at any point and retain the response for future review [45, 60], detect an improvement from DHI data [75] valued by patients. However, physiological data remained stable or became worse over time might result in patients' feelings of burden [75].

Delivery media means the types (eg, mobile phones or computers) and ownership (eg, private or public phones) of devices, and types of channels (eg, text message or videos) that used for providing health information or care services. Mobile technology limits the burden of usage on patients and maximizes clinical utility compared to web-based technology [41, 48]. Video-based technology enhanced patients communication with HCPs through giving patients experience of closeness [43, 65]. Patient preferences among delivery channels were varied and impacted by many factors (eg, functionality, credibility and accessibility) [60]. Some patients preferred smartphone-based app [53], others enjoyed using direct SMS text messaging [51]. Some participants were most comfortable with using their own personal devices [51, 53, 62, 70]. However, some patients preferred devices without personal identifiers [62].

#### Intervention Functionality

What kind of health information and care services are suitable for providing patients through the new digital health technology? To answer this question, intervention functionality needs to be considered. It refers to intervention goals, theoretical background, social support, intervention structure and performed quality. Intervention goals mean the intended use of DHI, including remote data monitoring [28, 41, 45, 48, 50, 51, 53, 55, 56, 58, 61, 63-65, 74-76], self-management support [28, 41, 42, 46, 51, 55, 56, 58, 69, 70, 75], health information provision and patient education [46, 48, 51, 54, 55, 60, 61, 63, 69, 82], individualized or timely feedback [42, 44, 48, 51, 55, 61, 62, 70, 75, 80], shared decision-making [41, 43, 44, 46, 48, 55, 56, 61, 65, 70]. Knowing that the data was being monitored, being able to remote consult HCPs, or receiving sufficient health information [28, 41, 45, 46, 55, 70] helped patients to better understand their health conditions and prepare for communication with HCPs, then obtain a sense of reassurance. DHI can lead to both self-care and health care dependency [41]. However, in some cases, patients felt a loss of independence as they were forced to share data with providers they may not want to [70] or under long-term video-based monitoring [40].

Theoretical background refers to the presence of underlying theory, including behaviour change techniques (BCTs), persuasive technology, and evidence-based interventions (EBIs), in the development of DHIs. DHIs with multiple BCTs appeared to be more effective [42, 61, 80] and higher patient reported satisfaction [48, 76] than those using fewer for supporting self-management of chronic disease. BCTs [42, 48, 52, 54, 58, 80] refer to varied categories, such as feedback, monitoring, goals and planning. Similarly, persuasive technology [42, 54, 74] was also used for improving patients’ behaviours, especially for people with diseases like type 2 diabetes where behavioural management is needed [74]. EBIs [54, 63, 78] were also recommended to increase patients’ credibility of DHIs.

Social support means the assistance in DHI available from HCPs, other patients, patients’ family or friends. The assistance can be emotional, informational, companionship supportive resources. Being able to interact with a human being was valued by patients [41]. The role of communication with a health coach or a peer might influence patient acceptance or adherence [52, 60, 63]. The increased regularly and continuous patient-to-physician interaction enabled by DHI gave patients a sense of reassurance and a peace of mind [41, 43, 45, 61, 63, 65, 66, 70, 74]. Connecting with peers helped them engage self-management [52, 74], enhance patient trust [82], and obtain better quality of life [40]. Patients enjoyed exchanging health information and advice with their family and friends through DHI [46, 60]. On the contrast, some patients reported they felt lacking physical human contact with their HCPs through remote communication [28, 43, 45, 46, 70, 79], and they worried interpersonal connections with HCPs were replaced by remote monitoring [28]. Additionally, being unable to contact their HCPs directly or obtain timely feedback resulted in increased patient anxiety [70].

Intervention structure refers to the intensity, frequency or duration of interventions. Structured interventions usually have fixed schedules to which patients are expected to adhere [41]. A structured format or regular weekly contact with HCPs helped patients create a sense of security [41, 45]. Longer duration might result in better health outcomes [55]. However, for those structured interventions were not tailored enough to patients’ individual symptoms and preference can be perceived as burden [45, 60]. Some patients experienced more stressful and anxiety due to the constant reminder of their symptoms [63, 70]. Therefore, in some cases, a flexible intervention rather than prefixed schedule was suggested [62].

Performed quality refers to how well the DHI works on the act of doing what is intended. System quality, interaction quality and information quality impacted patients perceived quality of whole DHIs which is defined as the user impression of the excellence of the digital health service [64]. Including the privacy and security [40, 41, 46, 47, 51, 53-55, 58, 60, 63, 64, 70, 76], reliability and credibility [40, 47, 54, 60, 64, 78], accuracy and validity [40, 41, 48, 51, 53, 70], availability and accessibility [40, 60, 62, 69], flexibility [47, 51, 64] of DHIs. Patients, especially who had a stigmatized health issue (eg, HIV), worried about their privacy [60]. Privacy notifications, security of device or health information, regulation compliance, and data storage can impact PEx [40, 46, 54, 55, 64]. The importance of the correct intervention dosage was highlighted [45, 48]. In some cases, patients, as well as professionals, questioned the accuracy of clinical assessments through DHI [41, 70]. If clinical indicators not be well defined or safely standardized might result in unnecessary stress to patients [63]. Owner’s credibility, maintenance, third party verification, research support, involvement of clinical experts in design process and empirical evidence for successful implementation impacted the credibility of DHIs [54, 64].

#### Intervention Interaction Design

Intervention interaction design contains personalized design, information design, navigation design, interface design, and design procedures. Personalized design refers to provide tailoring DHIs to the individuals according to their severity of disease or level of impairment and preferences on received time and place [40, 45, 51, 69]. Individualized feedback or personalized services [48, 54, 55, 58, 61, 62, 64, 74, 75, 78], tailored features [42, 48, 50, 51, 58, 69, 70, 76, 82], and customization [64] in DHI were valued by patients. Being able to choose the topic, content and language of received messages, select the timing and frequency of the delivered interventions were appreciated by patients [54, 55, 60, 69, 80]. Personalized design helped patients felt being taking seriously, promoted patient adherence, provided meaning in daily life [65, 69, 75]. Otherwise, patients felt a sense of impersonal [45].

Information design refers to the source, presentation, content and architecture of the delivered health information impacts patient adherence directly [69]. Some patients preferred receiving messages send from a reliable, trusted, credible source, but others with stigmatised health conditions preferred an unmarked sender regarding their privacy [60]. Multimedia messages [60, 78] (ie, text combined with relevant pictures or videos, and use of various font styles, sizes, and colours to highlight information), detailed and comprehensive information [48, 52, 63], diverse and keep updated information [60] were suggested to attract patients’ attention. Minimising information overload for patients should be considered [82]. Patients preferred short, concise, personalised, clear, and direct messages in a language they could understand, and with a motivational, friendly, encouraging, polite, respectful, congratulatory, personalised, upbeat, positive, humorous, and relatable tone [60, 67, 78]. A more formal or clinical language should be incorporated for some functions (description of pathologies) but more informal language for others (evaluation of conducts) if the users wish [64]. Medication information and warnings should be layered from basic to advanced information [46].

Navigation design is relevant to instruction manuals and extra user trainings that help users familiar with the technology and use it efficiently. Good navigation design and technical support helps users to understand the system and to alleviate errors [42, 74], increase their confidence and capability of operating technology [28]. Especially, for those patients who were unfamiliar with technology, or living in the rural settings, technical support is necessary for them in minimizing disparity of technical skills [40, 63, 69]. Technical assistance was reported as a facilitator by older patients and those with less experience using technology [46, 70]. Lack of clear navigation or instruction design [42, 48, 50, 52, 54, 64, 78] leads to a negative digital PEx.

Visual Design refers to the aesthetics, attractiveness and consistency of the appearances and screens of DHI. Visualized health data [75], tailored, attention-grabbing, simple and consistent layout design [42, 43, 67, 78], including appealing graphic presentation, pleasing and coherent colour scheme, high text quantity, suitable font size, striking button’s appearance and location, as well as appropriate interface size contributed to enhanced UX. On the contrast, unappealing user interfaces [40, 64], poorly crafted interface [74, 76], low visibility of the content [56] impeded quality of UX. Patients preferred to have a little smaller and watch-likes wearable health devices to make them more unobtrusive [50]. Bulkiness [50], non- portability [43] and small screen or font size [43, 64] of the monitoring devices were reported as negative features.

Design Procedures mean the design methodologies that used for developing DHIs. The user-centered design or human-centered design [28, 40, 46, 51, 58, 61, 62, 67, 70], inter-organisational collaboration [40, 72, 76], co-design or participatory development methodology [40, 46, 47, 51], as well as inclusive design [46, 77] can help to produce the multimodal, accessibility, adaptability, and usability of system. Involvement of multi-stakeholders and multi-disciplinary teams, including computer scientists, interaction designers, clinical professionals, and users or patients, in the early design stages was important [42, 45, 46, 51, 52, 58, 64, 76, 81].

#### Organizational Environment

Organizational environments refer to cost, health care providers, and health information systems. Cost means the amount of money or time that patients have to spend for buying the equipment, experiencing the treatment, or completing the tasks. Some patients appreciated to use DHIs since it is reducing the stress and burden of travel, cutting waiting time, and accessing quality care faster, then saving time and money [45, 60, 63, 73, 79]. For economic issues in DHI, they refer to start-up costs, ongoing costs, and costs related to loss of revenue [76]. In some cases, patients expressed concern over being liable for the cost of damage to the equipment [70], and on-going costs of remote monitoring [28]. Unrealistic financial reimbursement, higher cost relevant to Internet or equipment were the practical challenges of using DHI [46, 47, 60, 61, 70]. For time issues, some patients expressed they wanted to compete tasks at their own pace [69, 80], faster response [46, 60] and less time consuming [70]. They felt a sense of security as they can receive faster response [46, 51, 56], real-time feedback [48, 62, 65], or timely support [70]. Time consuming for daily monitoring or recharging devices, energy to complete “one more task”, or disruption to daily routine [45, 63, 64, 69], lack of timely feedback [50,70], and waiting times for digital health calls were too long [79] was perceived as a barrier.

Health care providers’ communication skills, professional ability and attitudes towards DHI impact PEx [57, 69]. Some patients expressed they want more HCPs in health care system [46, 79]. Clinician resistance appears to be particularly significant on the implementation of DHI [47], their opinion and approval influences whether patients welcome the DHI [50]. However, HCPs held the less positive views on telehealth due to it undermined their capacity and professional identity, resulted in less accurate clinical assessments, increased their workload, impeded communication with patients and leaded to overtreatment [41, 75]. Health information systems refer to the systems' compatibility, interoperability, integration, sustainability and completeness on an organizational level, as well as the clarity and transparency on accountability, workflow and data processing. Clarity on accountability [41, 47, 69, 75], transparent workflow [45, 51], as well as clear information on required stakeholders’ responsibility [51] was valued by patients. Both patients and HCPs need to be informed on who of them would be responsible for responding to out-of-range readings, and whether the data has been dealt with [45, 69, 75]. However, a review indicated patients actually do not have any power to make decision on their processes in the system [64]. Lack of compatibility and interoperability of the system with different mobile operating systems and terminals [40, 42, 56, 64, 67, 76], poor integration and working relationship between the service team [47, 51], lack of adequate installation [57, 69], connectivity issues between medical devices and mobile terminals [56], and limitation on scalability [76] often resulted in low quality of UX.

#### Physical Environment

Physical environments of patients can influence their experience of healthcare [51, 67, 76]. Being able to stay in a familiar and relaxing environment not restricted to the hospital setting [45, 50, 79] which can help patients focus on medical issues and might give them a sense of moving on and away from a clinical environment. Some patients felt more comfortable, in control, and in a safer place at home compared with being in hospital [44].

#### Social Environment

The absence of or inadequate supporting policies and legislation, lack of a plausible business case, unrealistic financial reimbursement, or lack of well-established sociotechnical infrastructure limited patient to adopt DHIs [47, 51, 76, 82].
